# Auranofin attenuates TOPBP1-mediated ATR replication stress response and improves chemotherapeutic response in breast tumor models

**DOI:** 10.1172/JCI180106

**Published:** 2025-12-15

**Authors:** Shuai Ma, Yingying Han, Rui Gu, Qi Chen, Qiushi Guo, Yuan Yue, Cheng Cao, Ling Liu, Zhenzhen Yang, Yan Qin, Ying Yang, Kai Zhang, Fei Liu, Lin Liu, Na Yang, Jihui Hao, Jie Yang, Zhi Yao, Xiaoyun Mao, Lei Shi

**Affiliations:** 1 Key Laboratory of Breast Cancer Prevention and Therapy (Ministry of Education), National Key Laboratory of Blood Science, Key Laboratory of Immune Microenvironment and Disease (Ministry of Education), The Province and Ministry Co-sponsored Collaborative Innovation Center for Medical Epigenetics, Tianjin Medical University Cancer Institute and Hospital, National Clinical Research Center for Cancer, Tianjin Medical University, Tianjin, China.; 2 Center for Reproduction and Genetics, Department of Obstetrics and Gynecology, The First Affiliated Hospital of University of Science and Technology of China (USTC), Division of Life Sciences and Medicine, USTC, Hefei, China.; 3 Core Facilities Center, Capital Medical University, Beijing, China.; 4 State Key Laboratory of Medicinal Chemical Biology, College of Pharmacy, Nankai University, Tianjin, China.; 5 Breast Surgery, The First Affiliated Hospital of China Medical University, Liaoning, China.

**Keywords:** Cell biology, Oncology, Therapeutics, Breast cancer, DNA repair

## Abstract

Genome instability is most commonly caused by replication stress, which also renders cancer cells extremely vulnerable once their response to replication stress is impeded. Topoisomerase II binding protein 1 (TOPBP1), an allosteric activator of ataxia telangiectasia and Rad3-related kinase (ATR), coordinates ATR in replication stress response and has emerged as a potential therapeutic target for tumors. Here, we identify auranofin, the FDA-approved drug for rheumatoid arthritis, as a lead compound capable of binding to the BRCT 7–8 domains and blocking TOPBP1 interaction with PHF8 and FANCJ. The liquid-liquid phase separation of TOPBP1 is also disrupted by auranofin. Through targeting these TOPBP1-nucleated molecular machineries, auranofin leads to an accumulation of replication defects by impairing ATR activation and attenuating replication protein A loading on perturbed replication forks, and it shows significant anti–breast tumor activity in combination with a PARP inhibitor. This study provides mechanistic insights into how auranofin challenges replication integrity and expands the application of this FDA-approved drug in breast tumor intervention.

## Abstract 2

We identified auranofin as an effective inhibitor of TOPBP1 and it elicits a synthetic vulnerability of breast tumor to PARP inhibitors by challenging replication integrity.

## Introduction

Replication stress response is a critical cellular defense mechanism that protects the integrity and stability of the genome (1, 2). Replication stress can arise from a variety of sources, including DNA damage, nucleotide depletion, or collisions between replication forks and other obstacles (1, 3). The master regulator of replication stress response is the protein kinase ataxia telangiectasia and Rad3-related (ATR), which coordinates the cellular response to replication stress by orchestrating a series of signaling events (4–6). Upon activation, ATR phosphorylates and activates multiple downstream effectors, such as the serine/threonine checkpoint kinase CHK1 and replication protein A (RPA), resulting in stabilization of replication forks, inhibition of cell cycle progression, and promotion of DNA repair (4, 7). By coordinating these molecular processes, ATR ensures that cells are well-equipped to respond to replication stress and have sufficient time to repair damaged DNA and maintain genome stability.

Aberrant activation or dysregulation of the ATR pathway has been implicated in various human diseases, including cancer (8, 9). Cancer cells often experience higher levels of replication stress due to their rapid and uncontrolled proliferation (10). Consequently, cancer cells rely heavily on the ATR pathway to cope with and tolerate replication stress, making ATR an attractive target for cancer therapy (8, 9). Numerous studies have demonstrated the effectiveness of ATR inhibitors in inducing synthetic lethality in cancer cells (11–14). In addition, the combination of ATR inhibitors with other DNA-damaging agents, such as chemotherapy or radiotherapy, has shown promising results in preclinical models and clinical trials (15–17). Although ATR inhibitors hold great promise for the treatment of cancer (18, 19), challenges remain, including the identification of reliable biomarkers to predict response to ATR inhibitors and concerns about toxic off-target effects in normal cells that undergo replication stress to a lesser extent.

Topoisomerase II binding protein 1 (TOPBP1) is a key allosteric regulator involved in the activation of ATR kinase at stalled or collapsed replication forks, where ssDNA is exposed and coated by RPA (4, 20). TOPBP1 stimulates ATR activity by undergoing liquid-liquid phase separation (LLPS) and forming a complex with ATR (4, 21–23). The recruitment of TOPBP1 to replication stress sites is mediated by the interaction of its conserved multi-BRCA1 C-terminal (BRCT) domains with phosphorylated or unphosphorylated protein clients (4, 24, 25). In contrast to the N-terminal BRCT domains 1 and 2 (N-terminal BRCT 1–2), which is responsible for TOPBP1-promoted DNA replication onset and fork protection (26, 27), BRCT 7–8 is only required for TOPBP1 to counteract replication stress by interacting with histone demethylase PHD finger protein 8 (PHF8) and BRCA1-associated C-terminal helicase/Fanconi anemia group J protein (BACH1/FANCJ) (28, 29). Aberrant expression of TOPBP1 has been observed in multiple malignancies, including breast, ovarian, lung, and colorectal tumors, and high TOPBP1 expression is correlated with poor prognosis and breast tumor aggressiveness (30–34), suggesting its potential as a tumor-promoting or prognostic marker. In addition, we reported that targeting BRCT 7–8 with GFP-oriented PHF8 fragment elicits a breast tumor–specific vulnerability to chemotherapeutics (34). These findings underscore the importance of developing or identifying small-molecule inhibitors against TOPBP1 BRCT 7-8 for chemotherapeutic treatment of breast tumors.

In this study, we identified auranofin, an FDA-approved anti–rheumatoid arthritis agent, as a TOPBP1 inhibitor with high potency in disrupting the interaction of TOPBP1 with PHF8 and FANCJ by docking to the hydrophobic pocket of BRCT 7–8. Auranofin was further found to impair the nuclear condensation of TOPBP1, attenuate the activation of ATR, and interfere with the loading of RPA, independent of its activity in generating ROS and inhibiting the ubiquitin-proteasome system (35–37). Importantly, when investigating the combination of auranofin with PARP inhibitor rucaparib, we observed a cancer cell–specific synthetic lethality of these drugs and a more pronounced breast tumor regression even in the presence of an effective ROS scavenger, underlying the potential efficacy of this combination strategy for tumor management. This study not only sheds light on the mechanism by which auranofin interferes with replication stress response and suppresses breast tumorigenesis but also highlights a specific vulnerability within breast tumors that can be exploited using PARP inhibitors.

## Results

### Auranofin directly targets TOPBP1 BRCT 7–8.

**Figure 1:**
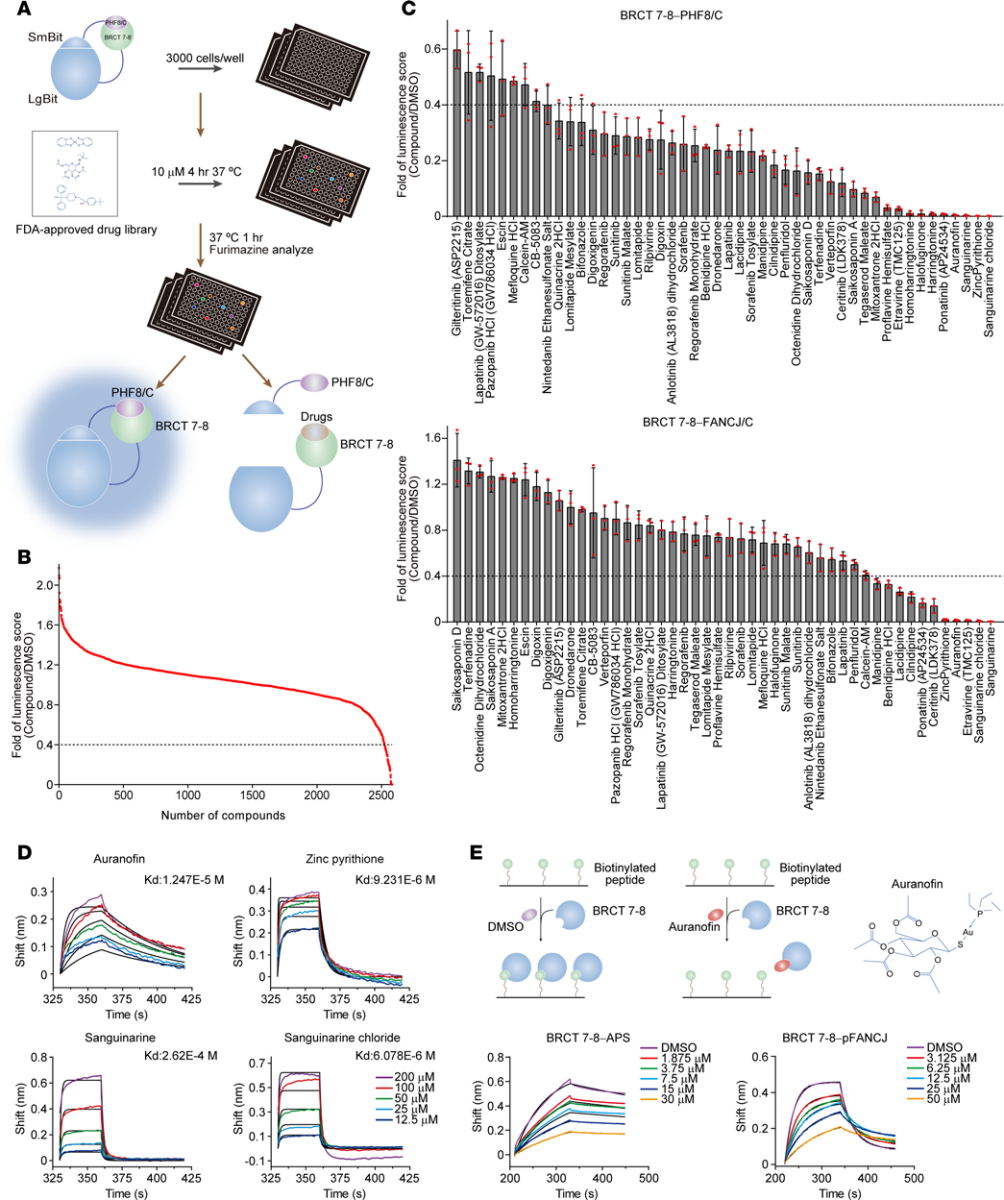
Auranofin directly targets TOPBP1 BRCT 7–8. (**A**) Schematic illustration of drug screening based on the NanoBiT system. HeLa cells stably expressing LgBiT-BRCT 7–8 and SmBiT-PHF8/C were treated with DMSO and 2,579 FDA-approved drugs (10 μM) for 4 hours followed by luminescent signal detection in live cells. (**B**) Summary of the initial drug screening. The luminescent signals in cells treated with different compounds were normalized with that of cells treated with DMSO; 47 compounds with normalized values lower than 0.4 (black line) were considered as potential candidates. (**C**) Secondary small-scale drug screening in HeLa cells stably expressing LgBiT-BRCT 7–8-SmBiT-PHF8/C and LgBiT-BRCT 7–8-SmBiT-FANCJ/C. These cells were cultured in the presence of 47 candidate compounds followed by an assessment of the luminescent signals. Calcein-AM was included as a positive control. Data are shown as mean ± SD. (**D**) Quantitation of the binding affinity between 4 candidate drugs and the His-tagged recombinant BRCT 7–8 purified from *E coli* cells by biolayer interferometry (BLI). BLI sensorgrams and the *K_D_* for each group are shown. Black lines are fitted curves; color traces are raw data. (**E**) BLI analysis of the inhibitory effect of auranofin on BRCT 7–8 binding to PHF8/APS peptide and phosphorylated Thr1133 containing peptide of FANCJ (pFANCJ). His-tagged BRCT 7–8 (1 μM) was preincubated with auranofin at the indicated concentrations before examining peptide-protein interactions. The experimental schemes and BLI sensorgrams are shown. Black lines are fitted curves; color traces are raw data.

By focusing on the BRCT 7–8 domains, we aimed to identify potential inhibitors of TOPBP1. First, we employed the NanoLuc Binary Technology–based (NanoBiT-based) protein-protein interaction system, a structural complementation reporter, to screen over 2,500 FDA-approved drugs for their ability to disrupt the binding of BRCT 7–8 to its cognate protein-ligand (Figure 1A). Specifically, the Large BiT (LgBiT) was fused to BRCT 7–8, and the complementary Small BiT (SmBiT) was fused to the C-terminal fragment of PHF8 (PHF8/C), which contains an acidic patch sequence (APS) capable of tightly interacting with BRCT 7–8 (28, 34). Upon stable integration of these 2 separate modules into cells, protein-protein interaction mediated by BRCT 7–8 and APS brings the split subunits (LgBiT and SmBiT) into proximity to form a functional enzyme that generates a bright luminescent signal. After 4 hours of incubation with individual drugs, luminescence was assessed in live cells, and an initial screening identified 47 drugs that exhibited a fold decrease of less than 0.4 in reducing the interaction between BRCT 7–8 and PHF8/C (Figure 1B). Using similar strategies, we then performed a second round of screening with the 47 drugs to test for their ability to inhibit the binding of BRCT 7–8 to the C-terminal region of FANCJ (FANCJ/C), whose phosphorylation at Thr1133 mediates the interaction (29, 38). This helped us to narrow down the number of drug candidates from 47 to 4 (Figure 1C). Calcein-acetoxymethyl ester (Calcein-AM), a known TOPBP1 inhibitor (30), was used as a positive control.

To identify small molecules that could directly target BRCT 7–8, we next employed biolayer interferometry (BLI), an optical biosensing technology that analyzes biomolecular interactions in real time, without the need for fluorescent labeling, to analyze the interactions between BRCT 7–8 and the 4 candidate drugs in vitro. The results showed that 3 of these drugs displayed a *K_D_* in the near micro-molar range in binding to the recombinant BRCT 7–8 purified from bacterial cells (Figure 1D). To further determine which one could functionally target BRCT 7–8, we measured the affinity change of BRCT 7–8 with APS peptide and phosphorylated FANCJ (pFANCJ) peptide by adding different concentrations of the drugs. Compared with the other two, BLI analysis showed that only auranofin, a gold (I)-containing phosphine compound, could effectively disrupt the interaction of BRCT 7–8 with the tested peptides (Figure 1E and Supplemental Figure 1, A and B; supplemental material available online with this article; https://doi.org/10.1172/JCI180106DS1). These findings suggest that auranofin has the potential to occupy the surfaces on BRCT 7–8 and block the interaction between BRCT 7–8 and both PHF8 and FANCJ.

### Auranofin disrupts the interaction of TOPBP1 with PHF8 and FANCJ.

**Figure 2:**
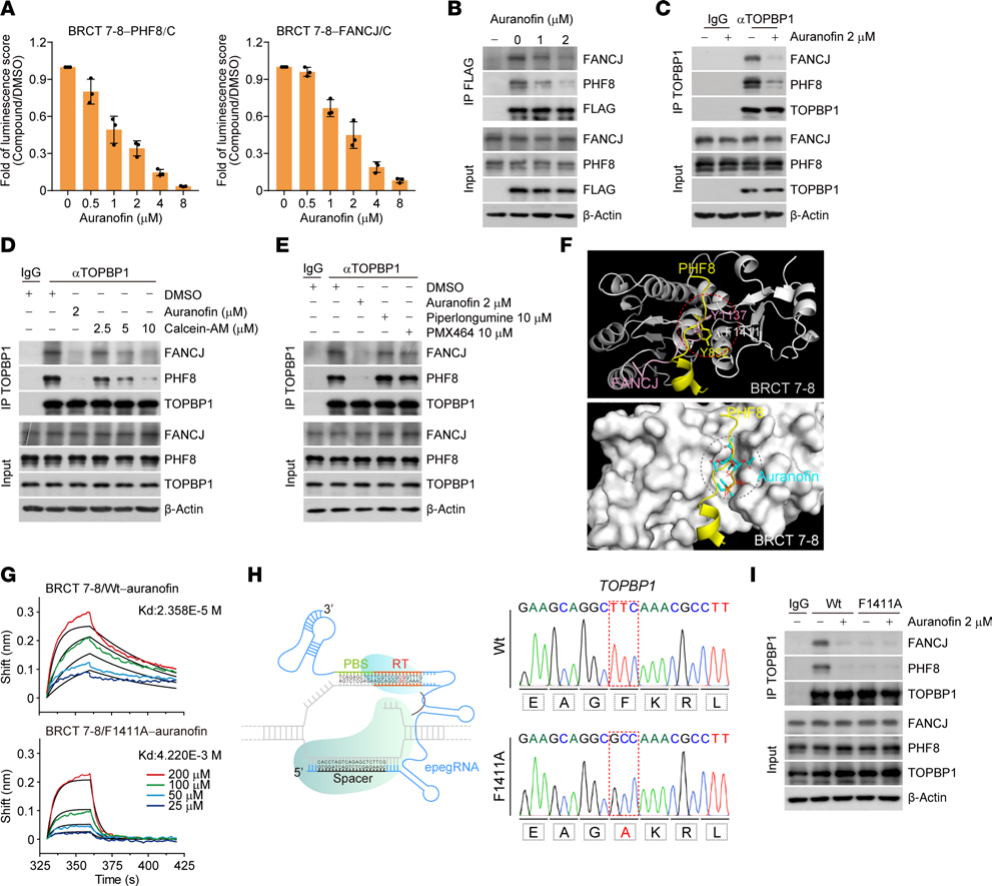
Auranofin disrupts the interaction of TOPBP1 with PHF8 and FANCJ. (**A**) Analysis of the interaction of BRCT 7–8 with PHF8/C and FANCJ/C by the NanoBiT system in HeLa cells treated with auranofin for 2 hours at the indicated concentrations. Data are shown as mean ± SD. (**B**) IP followed by immunoblotting with cellular extracts from FLAG-TOPBP1-expressing HeLa cells under 2 hours of treatment with auranofin at the indicated doses. (**C**) Co-IP analysis of the interactions of TOPBP1-PHF8 and TOPBP1-FANCJ with cellular extracts from HeLa cells treated with auranofin (2 μM, 2 hours). (**D**) Co-IP analysis of the interactions of TOPBP1-PHF8 and TOPBP1-FANCJ in HeLa cells cultured in the presence of auranofin (2 μM, 2 hours) or different doses of Calcein-AM (4 hours). (**E**) Co-IP analysis of the interactions of TOPBP1-PHF8 and TOPBP1-FANCJ in HeLa cells treated by auranofin (2 μM, 2 hours), PMX464 (10 μM, 4 hours), or piperlongumine (10 μM, 4 hours). (**F**) Predicted structure of auranofin docking to the hydrophobic pocket on BRCT 7–8. The hydrophobic pocket (dashed circle) and critical hydrophobic interactions based on crystal structures of BRCT 7–8-PHF8/APS (PDB ID: 7CMZ) and BRCT 7–8-pFANCJ (PDB ID: 3AL3) are shown. (**G**) BLI analysis of auranofin binding to recombinant BRCT 7–8/WT and BRCT 7–8/F1411A (*n* = 2). BLI sensorgrams and the *K_D_* for each group are shown. Black lines are fitted curves; color traces are raw data. (**H**) Schematic representation of the prime editing system and the representative sequencing results of F1411A-edited genomes from HeLa cells. The sequences of critical elements of the epegRNA are shown. (**I**) Co-IP analysis of the association of TOPBP1 with PHF8 and FANCJ in control and F1411A-edited HeLa cells treated with vehicle or auranofin (2 μM, 2 hours). All immunoblots were repeated at least twice and one of them is shown.

To verify the blockage activity of auranofin in vivo, we first titrated the inhibitory effect of auranofin with the NanoBiT system. The luminescence score in cells stably expressing LgBiT-BRCT7–8 and SmBiT-PHF8/C or SmBiT-FANCJ/C was decreased by auranofin in a dose-dependent manner (Figure 2A). Then, IP followed by immunoblotting revealed that auranofin markedly attenuated the interaction of FLAG-tagged TOPBP1 with PHF8 and FANCJ (Figure 2B). Similarly, auranofin was found to be an effective blocker of endogenous TOPBP1-PHF8 and TOPBP1-FANCJ interactions in HeLa cells cultured at a lower concentration of 2 μM (Figure 2C).

Importantly, auranofin elicited a much stronger inhibitory effect on TOPBP1 than Calcein-AM (Figure 2D). In addition, we found that auranofin acts independently of its canonical target, thioredoxin reductase (TrxR) (36, 37), as evidenced by the negligible effects of TrxR inhibitors such as piperlongumine and PMX464 on TOPBP1 binding to PHF8 and FANCJ (Figure 2E). We further excluded the possibility that auranofin’s impact on the molecular interactions of TOPBP1 is connected to mitochondrial permeability. This conclusion is supported by the observation that cyclosporine, which acts as a mitochondrial permeability blocker (39), did not affect the inhibitory role of auranofin on the interactions between TOPBP1 and either PHF8 or FANCJ (Supplemental Figure 2A).

To further understand the molecular mechanism of auranofin’s inhibitory activity, we performed a molecular docking assay. Consistent with its hydrophobicity (40, 41), auranofin was found to successfully engage the hydrophobic pocket of BRCT 7–8 (Figure 2F). Since the aromatic residue Phe1411 in this pocket is critical for PHF8 and FANCJ binding (28), we hypothesized that Phe1411 should be required for auranofin to target BRCT 7–8. To this end, Phe1411 was replaced by alanine, and WT BRCT 7–8 (BRCT 7–8/WT) and BRCT 7–8/F1411A were purified from bacterial cells. BLI analysis with these recombinant proteins revealed that alanine substitution (F1411A) markedly reduced the ability of auranofin to bind to BRCT 7–8 (Figure 2G and Supplemental Figure 2B). To test the importance of Phe1411 in auranofin engagement and thus protein complex deformation, a prime editing tool (42) was employed to modify genomes and generate F1411A-edited HeLa cells (Figure 2H). Of note, auranofin and F1411A mutation could disrupt TOPBP1-PHF8 and TOPBP1-FANCJ interactions to a similar extent, while auranofin failed to further challenge the formation of these protein complexes in F1411A-edited cells (Figure 2I). Because of the absence of cysteine residues around the hydrophobic pocket of BRCT 7–8 (Figure 2F), we thought that the action of auranofin toward BRCT 7–8 would not be based on covalent interactions induced by the thiol side chain in cysteines and dissociated gold ions of auranofin. Taken together, these data highly suggest that auranofin disrupts the interaction of TOPBP1 with PHF8 and FANCJ by competitively engaging the hydrophobic pocket of BRCT 7–8.

We consistently demonstrated that the formation of TOPBP1-POLQ complex, which also depends on BRCT 7–8 (43), and the recruitment of POLQ to double-strand break sites during mitosis, are susceptible to auranofin treatment (Supplemental Figure 2, C and D). The following evidence supports the molecular basis: (a) a comparison of the co-crystal structures with the AlphaFold-multimer-predicted structures indicates that, as with PHF8 and FANCJ (28, 38), the relatively conserved aromatic residue F1491 of POLQ forms a π-π stacking interaction with the F1411 residue in the hydrophobic pocket of BRCT 7–8 (Supplemental Figure 2E); (b) F1411A mutation markedly attenuated TOPBP1-POLQ interaction (Supplemental Figure 2F); (c) POLQ’s F1491 residue is essential for its binding to TOPBP1 and recruitment to mitotic double-strand breaks (Supplemental Figure 2, G and H). Given the conservation of the hydrophobic motif at the BRCT 7–8 interface among tandem BRCT domain–containing proteins such as TOPBP1, BRCA1, and ECT2 (44), we next investigated the specificity of auranofin toward these BRCTs. As shown in Supplemental Figure 2I, auranofin had minimal to no impact on the interaction between TOPBP1 BRCT 0–2 and 53BP1, RAD9, and HTATSF1 (45, 46), or between TOPBP1 BRCT 4–5 and BLM (47). Additionally, we found that BRCA1 and ECT2 could still interact effectively with FANCJ and RACGAP1 (48, 49) in the presence of auranofin (Supplemental Figure 2J). Utilizing a chemo-proteomics strategy with biotin-labeled auranofin, we identified TOPBP1 as one of the top candidate interactors (Supplemental Figure 2, K, M, and N, and Supplemental Table 1), and the other BRCT domain–containing proteins could not be captured by auranofin (Supplemental Figure 2L). These findings imply that auranofin may specifically target the BRCT 7–8 region of TOPBP1, likely involving specific residues beyond the hydrophobic motif.

### Auranofin impairs TOPBP1 recruitment and ATR activation.

**Figure 3:**
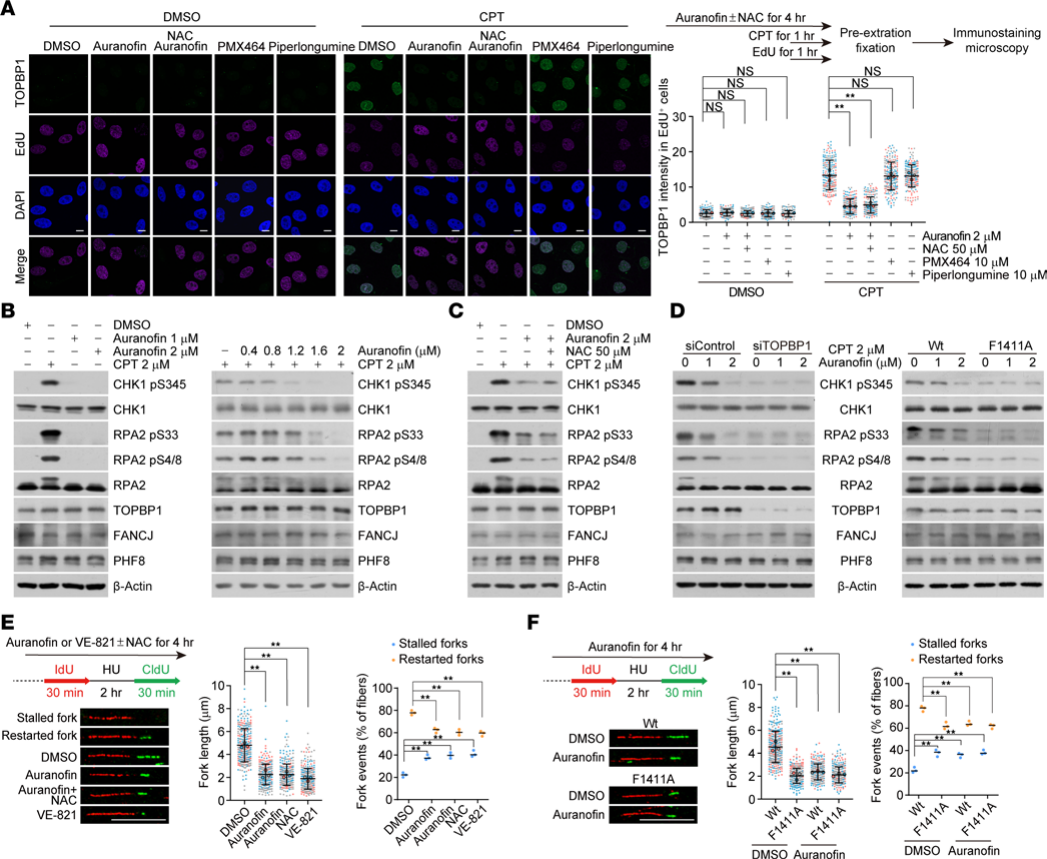
Auranofin impairs TOPBP1 recruitment and ATR activation. (**A**) Immunostaining and confocal microscopy analysis of TOPBP1 foci formation. U2OS cells were treated with auranofin (2 μM), PMX464 (10 μM), and piperlongumine (10 μM) in the absence or presence of NAC (50 μM) for 3 hours followed by additional 1 hour of camptothecin (CPT; 2 μM) challenge before pre-extraction and fixation. Cells undergoing active replication were labeled with EdU (10 μM, 1 hour), and the intensity of foci in EdU-positive cells was quantified and shown (*n* > 60). (**B** and **C**) Immunoblotting analysis of ATR kinase activity in U2OS cells under the treatment as indicated. (**D**) Analysis of ATR kinase activity by immunoblotting with cellular extracts from U2OS cells expressing TOPBP1 siRNA (left) and F1411A-edited HeLa cells (right) under the treatment as indicated. (**E**) Analysis of the fork lengths of restarted replication forks (CldU only) and the percentage of stalled (IdU only) or restarted forks (IdU-CldU) by DNA fiber assay in HeLa cells cultured in the presence of auranofin (2 μM), auranofin and NAC (50 μM), or VE-821 (10 μM). Cells were treated with hydroxyurea (HU; 2 mM) for 2 hours between IdU and CldU labeling to arrest replication forks (*n* > 90). (**F**) Analysis of fork lengths of restarted replication forks and the percentage of stalled or restarted forks by DNA fiber assay in control or F1411A-edited HeLa cells under vehicle or auranofin treatment (*n* > 100). Data are shown as mean ± SD (**A**, **E,** and **F**) from biological triplicate experiments. **P < 0.01; NS, not significant; 1-way ANOVA followed by Tukey’s multiple-comparison test (**A**, **E,** and **F**). Scale bars: 10 μm. All immunoblots were repeated at least twice and one of them is shown.

Considering that both PHF8 and FANCJ play important roles in TOPBP1 loading at sites of replication stress (28, 29), we thought that auranofin administration may lead to defective recruitment of TOPBP1 by disassembling protein complexes mediated by BRCT 7–8. Immunostaining followed by confocal microscopy analysis demonstrated that the foci formation of TOPBP1 was significantly compromised at camptothecin-induced replication stress sites in auranofin-treated cells (Figure 3A). Notably, these defects could not be overcome by ROS scavenger N-acetylcysteine (NAC), which could effectively counteract ROS-induced DNA damage (50) (Figure 3A and Supplemental Figure 3A). In addition, TOPBP1 foci formation was largely unaffected by TrxR inhibitors piperlongumine and PMX464 in camptothecin-treated cells (Figure 3A). By contrast, auranofin marginally altered the association of TOPBP1 with actively replicating chromatins in unstressed cells (Supplemental Figure 3B). These observations suggest that auranofin functionally inhibits TOPBP1 loading in the context of replication stress.

We next asked whether the auranofin-induced TOPBP1 loading defect suppresses ATR activity. First, we examined the phosphorylation level of CHK1 and RPA2, which are canonical substrates of ATR (20). Immunoblotting analysis showed that auranofin did not stimulate but impaired camptothecin-triggered CHK1 and RPA2 phosphorylation in a dose-dependent manner (Figure 3B), and this effect could not be ameliorated by NAC (Figure 3C). Importantly, we found that auranofin failed to further attenuate ATR activity in TOPBP1-depleted U2OS cells or F1411A-edited HeLa cells (Figure 3D). To further assess the inhibitory role of auranofin in ATR activation, we next examined replication fork integrity in the absence or presence of auranofin. Specifically, newly synthesized DNA was labeled with 5-iodo-2’-deoxyuridine (IdU), then treated with hydroxyurea, an inhibitor of the ribonucleotide reductase, and subsequently labeled with 5-chloro-2’-deoxyuridine (CldU) after removing hydroxyurea. Consistent with ATR inhibition as previously reported (51) and shown here, the addition of auranofin led to a significant reduction in replication strand lengths, an increased proportion of stalled replication forks, and a concomitant reduction of restarted forks (Figure 3E). Again, none of these effects were alleviated by NAC (Figure 3E). Similar phenotypes were observed in F1411A-edited cells, and no additive defects in replication integrity were observed upon auranofin administration (Figure 3F). Taken together, these data suggest that auranofin suppresses ATR activity by disrupting TOPBP1 recruitment.

### Auranofin dissolves TOPBP1 liquid-liquid condensate.

**Figure 4:**
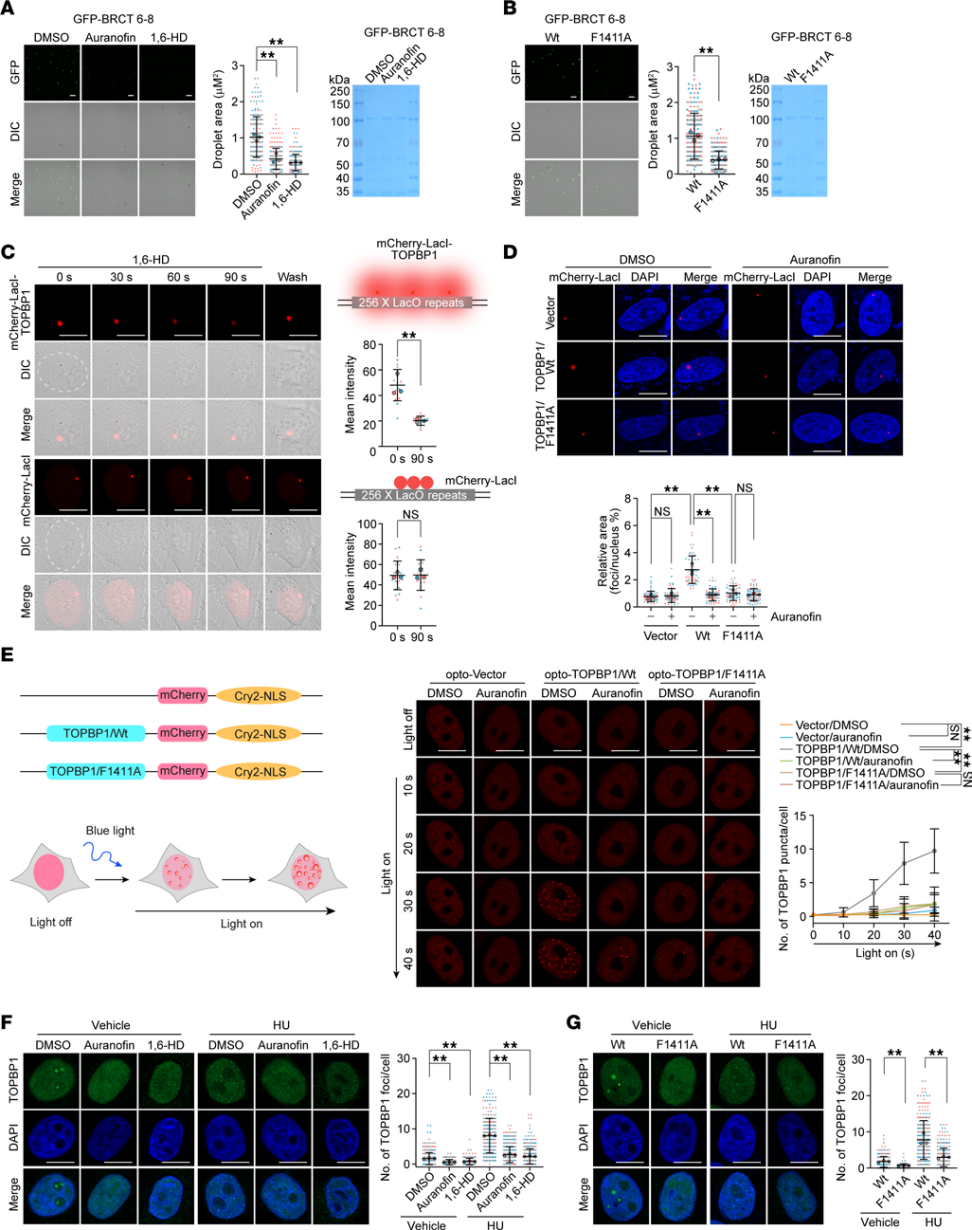
Auranofin dissolves TOPBP1 liquid-liquid condensate. (**A**) Liquid droplet formation of BRCT 6-8/WT observed by confocal and differential interference contrast (DIC) microscopy in the presence of vehicle, auranofin, and 1,6-hexanediol (1,6-HD) as indicated. The occupied areas of the droplets were quantified and shown. The GFP-tagged recombinant proteins purified from bacterial cells were examined by Coomassie brilliant blue (CBB) staining. (**B**) Representative micrographs and quantitative analysis of BRCT 6-8/WT and BRCT 6-8/F1411A droplet. The GFP-tagged recombinant proteins were examined by CBB staining. (**C**) Representative micrographs and quantitative analysis of the puncta intensity of mCherry-LacI-TOPBP1 and mCherry-LacI under 1,6-HD treatment (2.5%, 5 minutes) and removal (10 minutes later). U2OS cells stably integrated with 256 × LacO repeats were transfected with mCherry-LacI-TOPBP1 and mCherry-LacI, and the intensity of mCherry-marked puncta at each time point was quantified and shown (*n* ≥ 4). (**D**) Representative micrographs and quantitative analysis of the puncta intensity of mCherry-LacI-TOPBP1/WT, mCherry-LacI-TOPBP1/F1411A, and mCherry-LacI under auranofin treatment (*n* > 17). (**E**) Quantitative analysis of time-lapse opto-droplet formation of mCherry-Cry2–tagged proteins under vehicle or auranofin treatment after blue light activation (*n* > 15). A schematic of opto-droplet induction is shown. (**F**) Droplet formation of endogenous TOPBP1 under the indicated treatment. The number of larger puncta of nucleolar TOPBP1 under nonstressed conditions and nuclear-wide smaller TOPBP1 foci under hydroxyurea (HU) treatment in HeLa cells was quantified and shown (*n* > 60). (**G**) Representative micrographs and quantitative analysis of TOPBP1/WT and TOPBP1/F1411A droplets under the indicated treatment (*n* > 85). Data are shown as mean ± SD (**A**–**G**) from biological triplicate experiments. **P < 0.01; NS, not significant; 1-way ANOVA followed by Tukey’s multiple-comparison test (**A**, **D**, **F,** and **G**); unpaired 2-tailed *t* test with Welch’s correction for **B** and **C**; 2-way ANOVA for **E**. Scale bars: 5 μm for **A** and **B**; 10 μm for **C**–**G**.

It has been reported that TOPBP1 undergoes LLPS to self-assemble into micrometer-sized reaction centers to switch on ATR/CHK1 signaling (22). Therefore, we wondered whether auranofin is capable of interfering with TOPBP1 condensate formation. To this end, we first purified GFP-tagged BRCT 6–8, containing BRCT 6, ATR activation domain (AAD), and BRCT 7–8 domains. The recombinant BRCT 6–8 readily formed liquid droplets in vitro as reported (22), and they were, to a comparable extent, extremely sensitive to auranofin and 1,6-hexanediol (1,6-HD) (Figure 4A), which is widely used as a control to dissolve LLPS assemblies in phase-separation studies (52, 53). Furthermore, F1411A was found to markedly compromise the liquid-like structures of BRCT 6–8 (Figure 4B).

We then took advantage of a LacO-LacI interaction system (46, 54) to test TOPBP1 phase separation. Interestingly, mCherry-LacI-TOPBP1 was able to form much larger puncta than mCherry-LacI (Figure 4C). Similar to 1,6-HD, auranofin treatment significantly disengaged the condensate of mCherry-LacI-TOPBP1 but not mCherry-LacI (Figure 4C). In contrast to TOPBP1/WT, the smaller TOPBP1/F1411A puncta were resistant to 1,6-HD and auranofin (Figure 4D). Next, we used an optogenetic tool (55, 56) to evaluate the role of auranofin in suppressing TOPBP1 self-organizing biomolecular condensates. TOPBP1 was fused to arabidopsis-derived cryptochrome 2 (Cry2), an optogenetic protein that oligomerizes upon exposure to 488 nm light (Figure 4E). The results showed that TOPBP1/WT efficiently formed light-induced opto-droplets in living cells, whereas either F1411A mutation or auranofin treatment nearly abolished the clustering signal of opto-TOPBP1 (Figure 4E).

To further probe the effect of auranofin on biomolecular condensation of TOPBP1, we examined LLPS of endogenous TOPBP1. As reported (22), TOPBP1 forms distinctive puncta in the nucleolus under physiological conditions, whereas these droplets were disrupted by auranofin and 1,6-HD (Figure 4F). In response to hydroxyurea-induced replication stress, TOPBP1 exhibited numerous substructured clusters of nano-condensates, which were largely disintegrated by auranofin and 1,6-HD (Figure 4F). Likewise, a defective phase separation of TOPBP1 was observed in F1411A-edited cells (Figure 4G). Taken together, these results suggest that auranofin acts as a strong inhibitor of TOPBP1 liquid condensation.

Given the critical role of the low-complexity region in the AAD for TOPBP1 condensation (22), we propose that the intermolecular interaction between BRCT 7–8 and AAD, along with the weak AAD-AAD contacts, may collaboratively facilitate the formation of liquid-like droplet structures of TOPBP1. To test this hypothesis, we first analyzed the primary sequence of AAD (Supplemental Figure 4A). We found that residues Y989 and F1071 in TOPBP1 AAD correspond to Y852 of PHF8 (28) and Y1137 of FANCJ (38), both of which are critical for their interaction with BRCT 7–8. AlphaFold-multimer analysis indicated that either Y989 or F1071 formed a π-π stacking interaction with F1411 in the hydrophobic pocket of BRCT 7–8 (Supplemental Figure 4B). Furthermore, BLI analysis confirmed the essentiality of these residues in binding to BRCT 7–8 (Supplemental Figure 4, C and D), and auranofin effectively disrupted the interaction of BRCT 7–8 with either Y989- or F1071-containing peptide (Supplemental Figure 4E). Similar to F1071A (22), Y989A mutation also diminished TOPBP1’s ability to form condensates both in vitro and in vivo (Supplemental Figure 4, F and G). Remarkably, the double mutation produced a phenotype comparable to that observed with the TOPBP1 F1411A or treatment with auranofin (Supplemental Figure 4, F and G). These data suggest that BRCT 7–8–mediated intermolecular interactions of TOPBP1 are critical for its condensate formation, shedding light on the mechanisms behind auranofin-induced disruption of TOPBP1 condensation.

We next investigated the contribution of PHF8 and FANCJ to TOPBP1 LLPS formation. First, we demonstrated that PHF8 and FANCJ coalesced into prominent foci with mCherry-LacI-TOPBP1 around the LacO locus (Supplemental Figure 4, H–J). These foci were sensitive to treatment with 1,6-HD or auranofin (Supplemental Figure 4, H–J). Moreover, the depletion of either PHF8 or FANCJ resulted in a mild reduction in the size of TOPBP1 condensates (Supplemental Figure 4, K and L). These observations suggest that PHF8 and FANCJ could integrate into TOPBP1 condensates and contribute to its liquid condensation. Collectively, we propose that auranofin inhibits the assembly of TOPBP1 condensates by interrupting both the intermolecular interaction of TOPBP1 and the association of TOPBP1 with its protein partners.

### Auranofin prevents RPA loading to perturbed replication forks.

**Figure 5:**
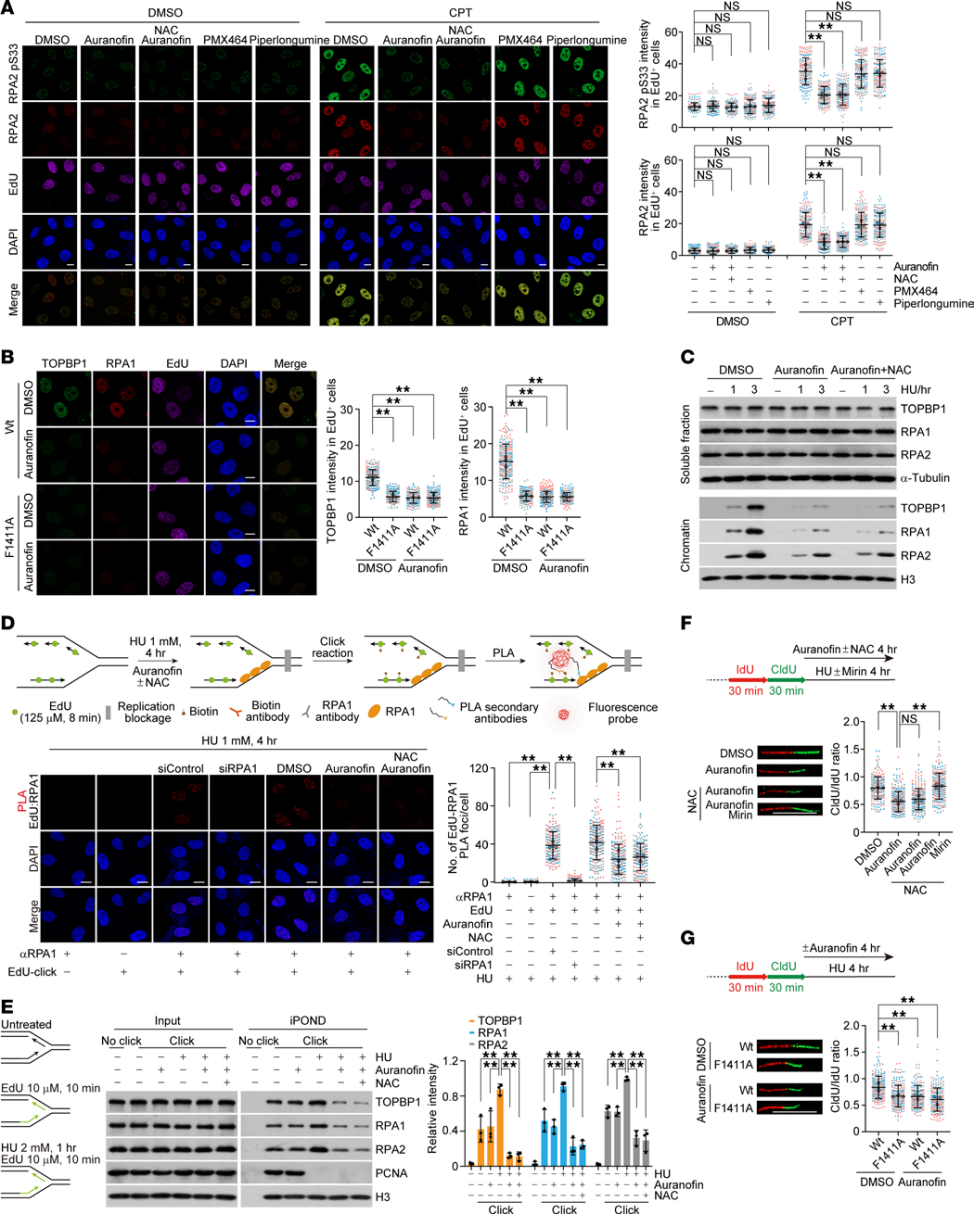
Auranofin prevents RPA loading to perturbed replication forks. (**A**) Immunostaining and confocal microscopy analysis of RPA2 and RPA2 pS33 foci formation. U2OS cells were treated with auranofin (2 μM), PMX464 (10 μM), and piperlongumine (10 μM) with or without NAC (50 μM) for 3 hours, followed by additional 1 hour of camptothecin (CPT; 2 μM) challenge before pre-extraction and fixation. The intensity of foci in EdU-positive cells was quantified and shown (*n* > 55). (**B**) Immunostaining and confocal microscopy analysis of TOPBP1 and RPA1 foci formation in CPT-treated control and F1411A-edited HeLa cells with or without auranofin. The intensity of foci in EdU-positive cells was quantified and shown (*n* > 60). (**C**) Immunoblotting analysis with soluble and chromatin fractions from HeLa cells under indicated treatment. (**D**) In situ analysis of protein interactions at DNA replication forks of interaction of RPA1 with nascent DNA (biotinylated EdU) in HeLa cells under indicated treatment. The proximity ligation assay foci number in each cell was quantified and shown (*n* > 70). (**E**) Isolation of proteins on nascent DNA analysis of proteins associated with blocked replication forks in HeLa cells under indicated treatments. (**F**) Replication fork stability was examined by DNA fiber assays in cells under indicated treatment. HeLa cells were sequentially labeled with IdU and CldU followed by hydroxyurea treatment in the absence or presence of mirin (100 μM, 4 hours). Ratios of CldU/IdU were quantified and shown (*n* > 60). (**G**) Replication fork stability examined by DNA fiber assays in control or F1411A-edited HeLa cells under indicated treatment (*n* > 60). Data are shown as mean ± SD (**A**, **B**, and **D**–**G**) from biological triplicate experiments. **P < 0.01; NS, not significant; 1-way ANOVA followed by Tukey’s multiple-comparison test (**A**, **B**, and **D–G**). Scale bars: 10 μm. All immunoblots were repeated at least twice and one of them is shown.

Given that TOPBP1-FANCJ interaction is required for the deposition of RPA onto ssDNA during replication stress (29) and that this interaction is sensitive to auranofin, we next sought to determine whether auranofin plays an adverse role in RPA loading. Immunofluorescence analysis showed that RPA2 as well as RPA2 pS33 foci formation was markedly compromised by auranofin, but not by piperlongumine and PMX 464, at camptothecin-induced replication stress sites, while NAC treatment had little to no effect on the auranofin-triggered RPA loading defect (Figure 5A). Similar results were obtained when examining the recruitment of RPA1 and TOPBP1 in F1411A-edited cells (Figure 5B). Consistently, auranofin caused a substantial decrease of chromatin-bound RPA and TOPBP1 in hydroxyurea-treated cells regardless of the absence or presence of NAC (Figure 5C). We then showed that neither FANCJ nor RPA could be captured by biotin-auranofin, and the formation of FANCJ-RPA complex (8) was resistant to auranofin (Supplemental Figure 5, A and B). Although auranofin has been reported to inhibit proteasome and proteasome-associated deubiquitinases UCHL5 and USP14 (35), it appeared that neither proteasome inhibition by bortezomib nor depletion of UCHL5 or USP14 apparently altered the foci formation of TOPBP1, RPA2, and RPA2 pS33, suggesting that auranofin acts independently of the ubiquitin-proteasome system in poisoning replication stress response (Supplemental Figure 5, C-E).

We next used in situ analysis of protein interactions at DNA replication forks to further assess whether auranofin blocks RPA loading at nascent replication forks (57). Briefly, HeLa cells were treated with hydroxyurea to induce fork stalling, and the interaction between RPA and nascent ssDNA was detected by proximity ligation assay (58) with primary antibodies against RPA1 and the biotinylated EdU, so that a productive proximity ligation assay signal of RPA1-EdU was produced only when RPA was localized within 40 nm of the nascent ssDNA. The results showed that RPA1 was localized at replication stress sites, whereas RPA1-EdU signal was significantly reduced under auranofin treatment without perturbation by NAC (Figure 5D). The same was true when the association of RPA1, RPA2, and TOPBP1 with nascent replication fork at blocked replicating chromatins was examined by isolation of proteins on nascent DNA assay (59) (Figure 5E). However, gel shift assays showed that the binding of recombinant RPA to ssDNA was not affected by auranofin, ruling out the possibility that auranofin could directly block RPA-ssDNA interaction (Supplemental Figure 5F). Nondenaturing BrdU staining demonstrated that auranofin addition led to a significant increase in the formation of BrdU foci upon replication stress (Supplemental Figure 5G). This suggests an increase in ssDNA accumulation, likely resulting from excessive firing of new origins due to ATR inactivation or exposure of ssDNA caused by the loss of RPA protection. Therefore, the defects in RPA loading in auranofin-treated cells could not be a result of a decrease in ssDNA generation. These results suggest that auranofin indirectly suppresses the loading of RPA onto ssDNA at replication stress sites, at least by deforming TOPBP1-scaffolded protein complexes.

DNA fiber analysis was then performed to assess fork deprotection caused by the auranofin-induced RPA loading defect at single-molecule resolution. HeLa cells were sequentially labeled with IdU and CldU and then treated with hydroxyurea for 4 hours. The lengths of DNA fiber tracks with continuous IdU and CldU were measured, and fork stability was reflected by the ratios of the lengths of adjacent CldU and IdU. The ratios approached unity under hydroxyurea treatment in control cells, indicating that the integrity of stalled forks was largely unaffected during replication stress. In contrast, the CldU/IdU ratios were significantly reduced in auranofin-treated cells (Figure 5F), implying a defect in stalled fork protection. This was prevented by addition of the nuclease inhibitor mirin (60) but not by NAC (Figure 5F). Consistently, nascent DNA degradation at stalled forks was observed in F1411A-edited cells, and there was no additive effect when these cells were cultured in the presence of auranofin (Figure 5G). These data suggest that auranofin compromises fork integrity in the presence of replication stress by inhibiting TOPBP1-FANCJ–mediated RPA loading.

### Auranofin confers vulnerability of breast cancer cells to chemotherapeutics.

**Figure 6:**
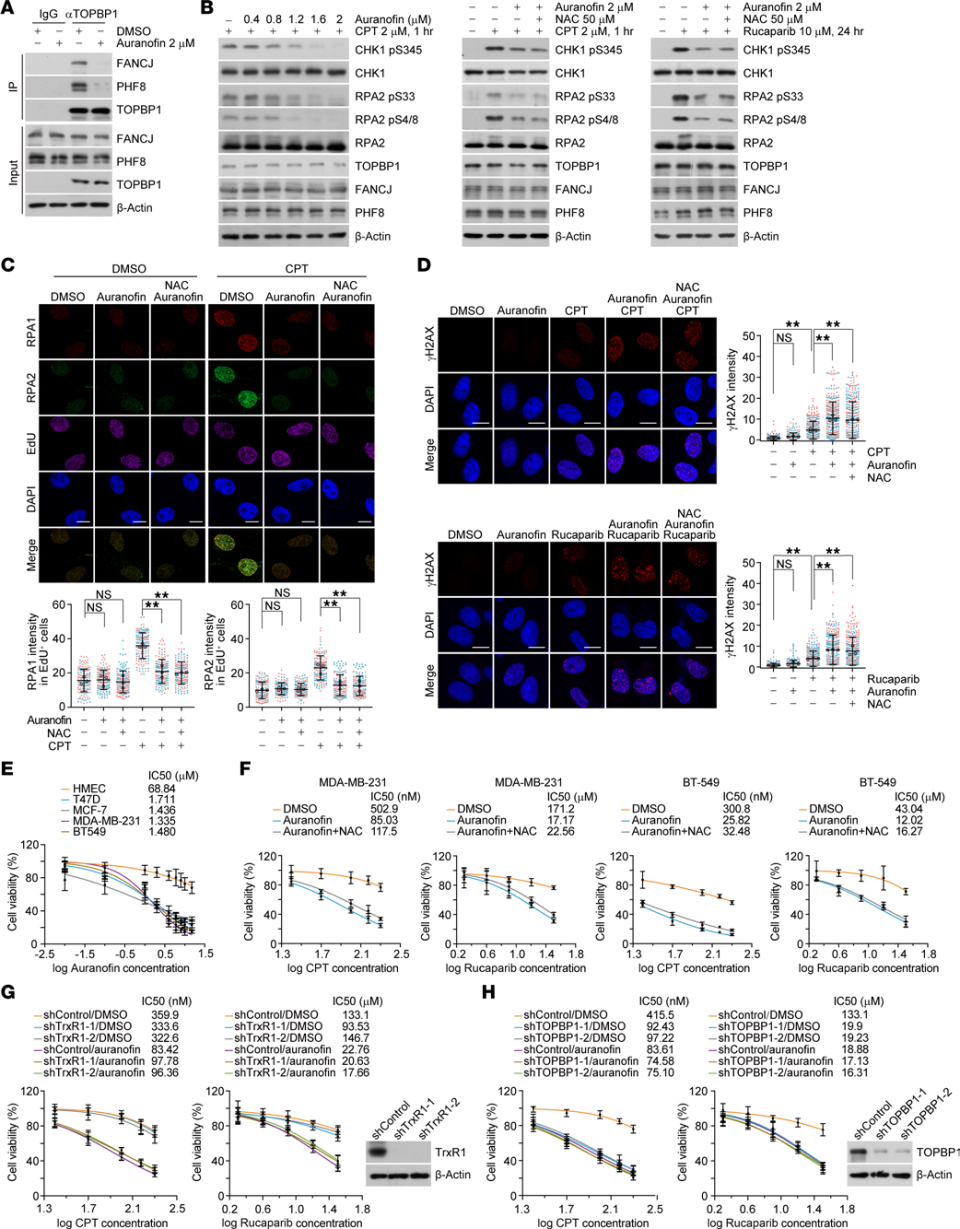
Auranofin confers vulnerability of breast cancer cells to chemotherapeutics. (**A**) Co-IP analysis of the interactions of TOPBP1-PHF8 and TOPBP1-FANCJ with cellular extracts from MDA-MB-231 cells treated with auranofin (2 μM, 2 hours). (**B**) Analysis of ATR kinase activity by immunoblotting with cellular extracts from MDA-MB-231 cells under the indicated treatments. (**C**) Representative micrographs and quantitative analysis of RPA1 and RPA2 foci formation. MDA-MB-231 cells were treated with auranofin (2 μM) in the absence or presence of NAC (50 μM) for 3 hours followed by additional 1 hour of camptothecin (CPT; 2 μM) challenge before pre-extraction and fixation. The intensity of foci in EdU-positive cells was quantified and shown (*n* > 40). (**D**) Representative micrographs and quantitative analysis of γH2AX foci formation in MDA-MB-231 cells under the indicated treatment. The intensity of γH2AX foci in each cell was quantified and shown (*n* > 50). (**E**) Survival analysis of human mammary epithelial cells and multiple breast cancer cells under different doses of auranofin treatment. The IC_50_ is calculated and shown. (**F**) Survival analysis of the synthetic lethality of auranofin (1.5 μM) and CPT or rucaparib in the absence or presence of NAC using MDA-MB-231 cells and BT-549 cells. (**G**) Survival analysis of the synthetic lethality of auranofin (1.5 μM) and CPT or rucaparib in TrxR-depleted MDA-MB-231 cells. The knockdown effect was confirmed by immunoblotting. (**H**) Survival analysis of the synthetic lethality of auranofin (1.5 μM) and CPT or rucaparib in TOPBP1-depleted MDA-MB-231 cells. The knockdown effect was confirmed by immunoblotting. Data are shown as mean ± SD (**C**, **D**, and **E**–**H**) from biological triplicate experiments. **P < 0.01; NS, not significant; 1-way ANOVA followed by Tukey’s multiple-comparison test for **C** and **D**; 2-way ANOVA for **E**–**H**. Scale bars: 10 μm. All immunoblots were repeated at least twice and one of them is shown.

In our previous study, we found that disrupting TOPBP1-PHF8 interaction suppresses breast tumorigenesis and creates a breast tumor–specific susceptibility to PARP inhibitor rucaparib and platinum drug cisplatin (34). Therefore, we investigated the potential antitumor role of auranofin in breast cancer cells. First, co-IP with cellular extracts collected from breast cancer cell MDA-MB-231 confirmed that auranofin impaired TOPBP1-PHF8 and TOPBP1-FANCJ interactions (Figure 6A). Next, immunoblotting analysis demonstrated that camptothecin- or rucaparib-induced ATR activation was dose dependently inhibited by auranofin (Figure 6B), and immunostaining followed by confocal microscopy revealed that auranofin disrupted RPA loading onto perturbed replication forks in MDA-MB-231 cells (Figure 6C). Consistently, auranofin dramatically increased γH2AX levels in cells treated with camptothecin or rucaparib (Figure 6D). Of note, we found that the inhibitory effect of auranofin on ATR activation, RPA loading, and genome stability was largely independent of the ROS system (61, 62), given that the antioxidant agent NAC failed to reverse any of the above phenotypes (Figure 6, B–D).

We next examined the effect of auranofin on cell survival and demonstrated that multiple breast cancer cells exhibited concentration-dependent vulnerability to auranofin treatment. In contrast, this effect was much less evident in human mammary epithelial cells (HMECs), highlighting the specific antitumor role of auranofin in breast cancer cells (Figure 6E). We then investigated the clinical relevance of auranofin in breast tumor chemotherapy and found that, unlike HMECs (Supplemental Figure 6, A and B), auranofin-treated MDA-MB-231 cells and BT-549 cells were more sensitive to camptothecin and rucaparib (Figure 6F). Importantly, this effect was unaffected by NAC (Figure 6F) and could not be phenocopied by TrxR depletion (Figure 6G). Thereby, auranofin is expected to increase the replication stress in cancer cells to a level that exceeds their tolerance threshold, leading to their selective death while sparing normal cells. Moreover, we found that auranofin did not elicit a more pronounced viability defect in TOPBP1-depleted breast cancer cells (Figure 6H). These findings suggest that auranofin synergizes with camptothecin or rucaparib by directly targeting TOPBP1 in breast cancer cells.

### Breast tumors are synthetically susceptible to the action of auranofin and rucaparib.

**Figure 7:**
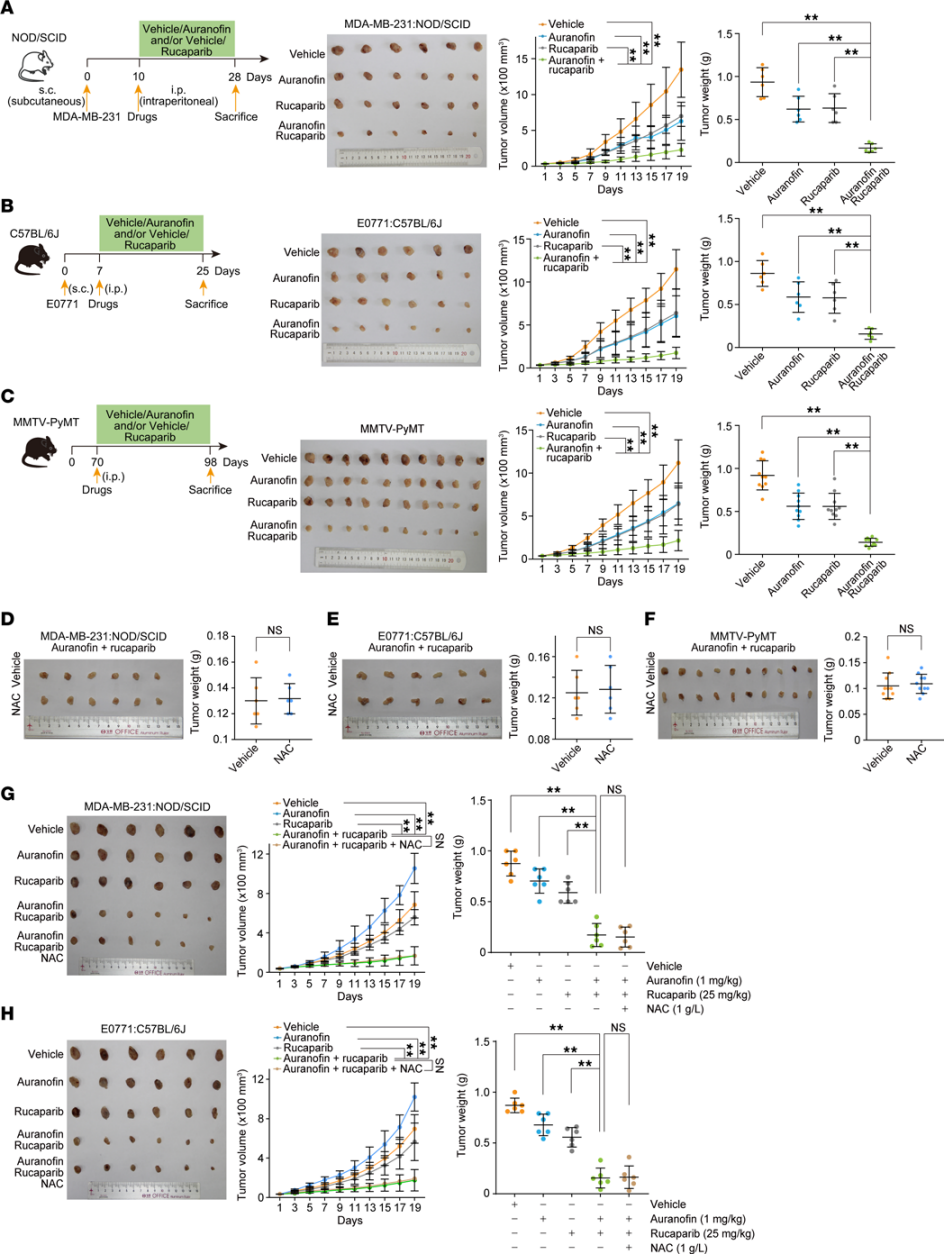
Breast tumor is synthetically susceptible to the action of auranofin and rucaparib. (**A**) Tumor size and weight of xenografts from MDA-MB-231 cells. NOD/SCID mice carrying tumors were treated with rucaparib (25 mg/kg), auranofin (5 mg/kg), or both every 2 days. The experimental scheme is shown. (**B**) Tumor size and weight of xenografts from E0771 cells. C57BL/6J mice carrying tumors were treated with rucaparib (25 mg/kg), auranofin (5 mg/kg), or both every 2 days. The experimental scheme is shown. (**C**) Tumor size and weight of genetically engineered mammary gland tumors. PyMT mice carrying tumors were treated with rucaparib (25 mg/kg), auranofin (5 mg/kg), or both every 2 days. The experimental scheme is shown. (**D**–**F**) Tumor weight of MDA-MB-231 cells (**D**), E0771 cells (**E**), and genetically engineered mammary gland tumors (**F**) under combinatorial auranofin and rucaparib treatment in the absence or presence of NAC (1 g/L in feeding water). (**G**) Tumor size and weight of xenografts from MDA-MB-231 cells. NOD/SCID mice carrying tumors were treated with rucaparib (25 mg/kg), auranofin (1 mg/kg), or both in the absence or presence of NAC every 2 days. (**H**) Tumor size and weight of xenografts from E0771 cells. C57BL/6J mice carrying tumors were treated with rucaparib (25 mg/kg), auranofin (1 mg/kg), or both every 2 days in the absence or presence of NAC (1 g/L). Data are shown as mean ± SD (**A**–**H**). **P < 0.01; NS, not significant; 2-way ANOVA for the left panels of **A**–**C**, **G**, and **H**; 1-way ANOVA followed by Tukey’s multiple-comparison test for the right panels of **A**–**C**, **G**, and **H**; unpaired 2-tailed *t* test with Welch’s correction (**D**–**F**).

To further investigate the antitumor activity of auranofin and its synthetic lethality with rucaparib, we established MDA-MB-231 xenografts in NOD/SCID mice and intraperitoneally injected vehicle, auranofin, rucaparib, or auranofin together with rucaparib. To circumvent the inhibitory effect of auranofin on TrxR, a low dose of auranofin was administered at 5 mg/kg. The results showed that auranofin and rucaparib alone mildly inhibited xenograft growth, while the combination of auranofin and rucaparib significantly suppressed tumor development (Figure 7A). Next, we injected E0771 cells, a murine breast cancer cell line on a C57BL/6J background, into the mammary gland of C57BL/6J mice to investigate the synergistic lethality of auranofin and rucaparib. Consistently, coadministration of these drugs resulted in a remarkable regression of E0771 tumors compared with each drug alone (Figure 7B).

We then used the mammary-specific polyomavirus middle T antigen overexpression mouse model (MMTV-PyMT), which has similar molecular and histological progression as human breast tumors, to confirm the antitumor function of auranofin. The results showed that auranofin significantly sensitized mammary gland tumors to rucaparib treatment (Figure 7C). It appeared that ROS-induced ferroptosis was unlikely to be involved in this process because low-dose auranofin administration only attenuated iron overload but not ferroptosis in mice (63). At the same time, we noticed that the growth defects of the 3 types of breast tumor could not be ameliorated by NAC administration, further suggesting the synergistic effect independent of causing higher levels of ROS (Figure 7, D–F). The body weight of mice under each treatment was essentially unchanged in the above experiments using different mouse tumor models (Supplemental Figure 7, A–F).

Moreover, auranofin showed a dose-dependent effect on E0771 tumors, with nondetectable toxicity indicated by changes in mouse weight, intestinal epithelium structure and proliferation, and blood parameters (Supplemental Figure 7, G–J). Despite the profound tumor regression and minimal toxicity observed in mice with the auranofin (5 mg/kg) and rucaparib combination (Supplemental Figure 7, G-J), we proceeded to investigate whether a clinically relevant dosage of auranofin (1 mg/kg administered every 2 days equivalent to 6.5 mg daily for a 65 kg patient by allometric scaling) could produce a synergistic antitumor effect with rucaparib. The results showed that this drug combination strategy led to MDA-MB-231 or E0771 tumor suppression almost comparable to that observed at a dose of 5 mg/kg auranofin (Figure 7, G and H). Meanwhile, these outcomes exhibited resilience to NAC treatment (Figure 7, G and H). Taken together, these findings highlight the translational potential of combining auranofin and rucaparib for treating breast tumors.

## Discussion

Auranofin, an FDA-approved drug used primarily for the management of rheumatoid arthritis, has garnered significant interest due to its potential role in cancer treatment (36, 40). It is classified as a small-molecule drug that has shown promising antitumor properties through its ability to disrupt various cellular processes, including reduction/oxidation (redox), proteasome-mediated protein degradation, and protein stability regulated by proteasome-associated deubiquitination (35, 40). Here, we report that auranofin can target TOPBP1 BRCT 7–8, thereby disrupting TOPBP1-nucleated molecular machineries, including protein complex formation and TOPBP1 liquid condensation, to suppress replication stress response in a manner independent of the known auranofin-regulated signaling pathways. This feature is further exploited for breast tumor intervention when coadministered with PARP inhibitor rucaparib, which is commonly used in the treatment of breast and ovarian tumors harboring BRCA mutations.

In contrast to the multifunctional roles of BRCT 1–2 (26, 27), BRCT 7–8 preferentially facilitates the recruitment of TOPBP1 at sites of replication stress (28, 29). This specificity positions BRCT 7–8 as a more promising target for drug intervention. Auranofin appears to bind to a hydrophobic pocket at the interface of the 2 consecutive domains of BRCT 7–8, a feature that is structurally conserved across tandem BRCT domains (64). This raises an important question regarding whether auranofin specifically disrupts the protein interactions associated with TOPBP1’s BRCT 7–8, or if it can also interfere with other interactions involving tandem BRCT domains (44). The observations that auranofin had minimal influence on protein complex formation directed by BRCT 0–2 or BRCT 4–5 of TOPBP1 and BRCT tandem domains from other proteins suggest auranofin may selectively target BRCT 7–8 of TOPBP1, likely involving domain-specific residues beyond the hydrophobic motif of tandem BRCTs in the interaction. Resolving the crystal structure of auranofin and BRCT 7–8 is crucial for understanding its mechanism of action. Although it remains to be determined whether other candidates identified by chemoproteomics truly interact with auranofin or if their presence is due to nonspecific binding, our findings, at least, support the argument that TOPBP1 BRCT 7–8 acts as an on-target effector of auranofin in blocking replication stress response. We also demonstrated that the effect of auranofin, ranging from TOPBP1 recruitment and ATR activation to RPA loading and fork protection, largely appears to operate independently of its influence on redox enzymes and the ubiquitin-proteasome system. However, we could not entirely rule out the possibility that long-term auranofin treatment will affect these pathways, thus accounting for its synergy with PARP inhibition in tumor regression. Similarly, the influence of auranofin on the disassembly of the TOPBP1-POLQ complex (43), which repairs mitotic double-strand breaks, should not be overlooked and requires further exploration.

In cells, TOPBP1 exists in 2 main forms: one is chromatin-bound and resistant to pre-extraction, which emerges during the early stages of replication stress (25), and the other manifests as a liquid-like condensation around chromatin, which is susceptible to pre-extraction and peaks at a later stage for ATR signal amplification (22, 65, 66). Given the essential function of BRCT 7–8 in recruiting TOPBP1 to damaged chromatin, as we previously reported (28), and its role in subsequently promoting liquid condensation, as we reported here, along with the specific interaction between auranofin and BRCT 7–8, it appears that auranofin interferes with both the chromatin-binding ability of TOPBP1 and its bio-condensate formation. Although the intermolecular interactions mediated by BRCT 7–8 and the AAD region are essential for TOPBP1 condensation, further research is needed to differentiate the roles of BRCT 7–8 from the low complexity or intrinsically disordered regions of AAD in the formation of TOPBP1 liquid condensates. In addition to interrupting the intermolecular interaction of TOPBP1, auranofin appears to disrupt the formation of TOPBP1 condensates by blocking the association of PHF8 and FANCJ with BRCT 7–8. Yet, the molecular mechanism and biological significance of PHF8 and FANCJ in promoting TOPBP1 condensation during replication stress response warrant further investigation.

During tumorigenesis, abnormal LLPS can lead to the formation of protein or RNA droplets that concentrate or sequester critical regulators of cell growth, signaling, and DNA repair (56, 67–69). These structures can alter the spatial organization and dynamics of cellular components, resulting in gain or loss of function of cellular processes. During breast tumorigenesis, highly expressed TOPBP1 may help cancer cells efficiently cope with the sustained replication stress by compartmentalizing itself and associated factors around ATR. It is reasonable to hypothesize that targeting and modulating TOPBP1 LLPS could provide important ways to inhibit TOPBP1-driven ATR activity and enhance chemotherapeutic efficacy. We herein provide a prototype strategy to prevent TOPBP1 condensate assembly for innovative therapeutic interventions, and further studies are needed to fully exploit and utilize the potential of TOPBP1 LLPS in tumor management.

So far, 2 small molecules, Calcein-AM and 5D4, have been identified as inhibitors of TOPBP1 by binding to BRCT 7–8 (30, 70). Since advancing an FDA-approved drug to clinical trials would be expeditious compared with novel compounds, auranofin stands out as a more promising candidate than Calcein-AM and 5D4 for cancer therapy. Nevertheless, additional research and clinical trials are essential to thoroughly evaluate its efficacy, safety, and long-term outcomes in combination with PARP inhibitors. Auranofin has been reported to synergize with PARP inhibitor olaparib to induce cell death in p53 mutant lung and pancreatic tumors (71). However, the activity of auranofin in these 2 types of malignancies is mediated by TrxR inhibition–induced ROS accumulation. Interestingly, auranofin induces cell death of T cell acute lymphoblastic leukemia by inhibiting dioxygenases TET1 instead of TrxR (72). These studies, together with our findings, suggest a tumor type–dependent target selection of auranofin, and further investigation is required to understand the precise mechanisms of auranofin action and potential off-target effects. In addition to being used in conjunction with rucaparib for the treatment of breast tumors, auranofin holds great promise as a repurposed drug for targeted cancer therapy due to its effective inhibition of replication stress response. This is particularly relevant for malignancies with synthetic lethal biomarkers, such as microsatellite instability or ARID1A deficiency, which could be preclinically targeted by ATR inhibitors (11, 12).

## Methods

### Sex as a biological variable.

All animal experiments used only female mice because the vast majority of breast cancers occur in women.

### Cell culture.

HeLa, U2OS, MDA-MB-231, T47D, MCF-7, BT549, HMEC, E0771, and HEK293T cells were purchased from ATCC and cultured under the manufacturer’s instructions. LacO-LacI U2OS cells were provided by Roger Greenberg (University of Pennsylvania, Philadelphia, Pennsylvania). All of the cells were authenticated by examination of morphology and growth characteristics and confirmed to be mycoplasma-free.

### NanoBiT assays.

NanoBiT assays were performed in HeLa cells stably expressing LgBit-BRCT 7–8 and SmBit-PHF8/C or LgBit-BRCT 7–8 and SmBit-FANCJ/C. Approximately 3,000 cells were seeded in each well of the 96-well plate. After 24 hours, the cells were treated with different drugs for 4 hours. The activity of nano-luciferase was measured according to the manufacturer’s instructions using Nano-Glo Live Cell Assay System (Promega, N2012) and Synergy plate reader (BioTek Instruments).

### BLI.

The BLI assays were performed using Octet RED96 (ForteBio). For binary binding assays, all the purified proteins were first biotinylated by EZ-Link NHS-Biotin (Thermo Fisher Scientific) according to the manufacturer’s instructions, and then were loaded onto SSA biosensors (ForteBio). Average saturation response levels of 15 nm were reached in 15 minutes for all the proteins. Sensors with proteins were washed in assay buffer (PBS with 0.1% BSA, 0.02% Tween-20, and 1% DMSO) for 10 minutes to establish stable baselines before starting association-dissociation cycles with tested compounds and DMSO as references. Raw kinetic data were processed in the data analysis software provided by the manufacturer using double reference subtraction in which both DMSO-only reference and inactive reference were subtracted. A 1:1 binding model was used to fit the association and dissociation rates. Equilibrium *K_D_* values were calculated from the ratio of K_off_ to K_on_. In competitive assays, PBS with 0.1% BSA, 0.02% Tween-20, and 1% DMSO was used as the assay buffer. Biotin-APS (GACFKDAEYIYPSLESDDDDPA), biotin-pFANCJ (EAEDESIYF(pT)PELYDPEDT), biotin-Y989 (ECKHLPESLY_989_PHTYN), and biotin-F1071 (TLEMRENF_1071_QKQLQEI) were immobilized onto the surface of biosensors. The association was conducted with binary complex (protein preincubated with a serial dilution of compounds) followed by 2 minutes of dissociation in assay buffer. A 1:1 binding model with local fit was used to determine the kinetic parameters.

### Generation of TOPBP1/F1411A-edited cells.

HeLa cells were transfected at approximately 60% confluency with 3 mg PE3, 1 mg epegRNA, and 0.4 mg nicking sgRNA plasmids. Cells were cultured for 3 days after transfection, after which a single colony was sorted by flow cytometry. When cells reached confluency, genomic DNA was extracted, followed by PCR amplification and DNA sequencing. The sequences of guide RNAs are provided in Supplemental Table 2.

### RNA interference.

All siRNA transfections were performed using Lipofectamine RNAi MAX (Invitrogen) following the manufacturer’s recommendations. The final concentration of the siRNA molecules was 5 nM, and cells were harvested 72 hours later. Control siRNA (ON-TARGETplus Non-Targeting Pool, D-001810-10) was purchased from Dharmacon, and the other siRNAs were chemically synthesized by Sigma-Aldrich. The siRNA and shRNA sequences are listed in Supplemental Tables 3 and 4.

### qRT-PCR.

Total cellular RNA was isolated with TRIzol reagent (Invitrogen) and used for first strand cDNA synthesis with the Reverse Transcription System (Roche). Quantitation of all gene transcripts was done by qPCR using a Power SYBR Green PCR Master Mix (Roche) and an ABI PRISM 7500 sequence detection system (Applied Biosystems). The qRT-PCR primers are listed in Supplemental Table 5.

### Immunofluorescence.

Cells were seeded on glass coverslips (BD Biosciences), fixed with 4% paraformaldehyde, and permeabilized with 0.2% Triton X-100 in PBS. Samples were blocked in 5% donkey serum in the presence of 0.1% Triton X-100 and stained with the appropriate primary and secondary antibodies coupled to Alexa Fluor 488, 594, or 647 (Invitrogen) (See supplemental materials for specific antibodies used.). To avoid bleed-through effects in double-staining experiments, each dye was scanned independently in a multitracking mode. When inspecting nuclear-wide dispersed RPA2, RPA1, TOPBP1, BrdU, or RPA2 pS33 foci, cells were pretreated with 0.5% Triton X-100 for 5 minutes on ice to extract non-chromatin fractions and fixed with 3% paraformaldehyde and 2% sucrose for 15 minutes at room temperature. Cells were then permeabilized with 0.5% Triton X-100 for 5 minutes on ice and incubated in blocking buffer (0.1% Triton X-100 and 5% donkey serum in PBS) for 1 hour at room temperature. For mitotic foci staining, cells treated with nocodazole (100 ng/mL, 16 hours) were irradiated (1 Gy) and fixed with ice-cold methanol for 8 minutes, then permeabilized with 0.2% Triton X-100 for 5 minutes. For S-phase discrimination, cells were pulsed with 10 μM EdU at 37°C for 1 hour before fixation. Incorporated EdU was click-labeled using keyFluor 647-azide (Keygen Technologies) according to the manufacturer’s instructions. For visualization of 8-oxoG in situ, cells on coverslips were fixed in acetone/methanol (1:1) and air dried. Cells were hydrated for 15 minutes in PBS, and DNA was denatured by incubating cells in 1.5 M HCl for 15 minutes at room temperature. Cells were washed 3 times in PBS and neutralized with 1 M Tris-base for 7 minutes before proceeding to the immunostaining protocol.

### In vitro phase separation assay.

Recombinant proteins (10 μM) in 15 mL buffer containing 20 mM Tris-HCl, pH 7.0, 1 mM dithiothreitol, and 10% PEG-8000 treated with auranofin (2 μM, 5 minutes) or 1,6-HD (2.5%, 5 minutes) were loaded onto a glass slide, covered with a coverslip, and imaged using a Zeiss LSM 900 confocal microscope system.

### Opto-TOPBP1 activation.

Cells were plated at around 70% confluency in DMEM. The plasmids encoding different TOPBP1 variants were transfected into HeLa cells. At 48 hours after transfection, cells were covered with aluminum foil to block light, and droplet formation was induced on a Zeiss LSM 900 microscope with 488 nm light pulses (12% of the full power). mCherry fluorescence was stimulated with 561 nm light on the microscope with a 63× oil objective, and time-lapse images were captured at 10-second intervals for 40 seconds. Puncta numbers in cells expressing different mCherry-Cry2-NLS-tagged TOPBP1 variants or empty controls were quantified.

### DNA fiber assay.

To check fork stability, cells were first labeled with IdU (25 μM) for 30 minutes, washed twice with media, and labeled with CldU (200 μM) for 30 minutes. After washing, cells were incubated with hydroxyurea with or without auranofin, NAC, and mirin. For examining the restart efficiency of stalled replication forks, cells were labeled with IdU (25 μM) for 30 minutes and then washed twice with media. After washing, cells were treated with hydroxyurea (2 mM) for 2 hours. Cells were then recovered in fresh medium with CldU (200 μM) for 30 minutes. Cells were then trypsinized and resuspended in PBS to 7 × 10^5^ cells/mL. Then, 2 μL of cells was mixed with 10 μL of lysis buffer (200 mM Tris-HCl, pH 7.4; 50 mM EDTA; and 0.5% SDS) on a clean glass slide. After 2 minutes of incubation, the slides were tilted at 15° to the horizontal, allowing the lysate to slowly flow down along the slide. The slides were then air-dried, fixed in 3:1 methanol/acetic acid, and treated with 2.5 M HCl for 80 minutes. The slides were then blocked in blocking buffer (5% BSA in PBS) for 30 minutes and incubated with anti-BrdU antibodies in blocking buffer overnight (See supplemental materials for specific antibodies used.). After washing, secondary antibodies coupled to Alexa Fluor 488 and 594 were diluted in PBS containing 5% BSA and incubated with cells at room temperature for 1 hour. The slides were then washed 3 times with PBS. After washing, cells were mounted with an antifade solution and visualized under a Zeiss LSM 900 fluorescence microscope. The length of all discrete fibers was measured by using ImageJ (NIH) software.

### Isolation of proteins on nascent DNA.

In brief, approximately 2 × 10^8^ to 3 × 10^8^ cells were labeled with 10 μM EdU (Thermo Fisher Scientific) for 10 minutes to detect nascent forks. For stalled forks, cells were treated with hydroxyurea for 1 hour in the continued presence of EdU. The harvested cells were then fixed with 1% formaldehyde in PBS solution for 20 minutes at room temperature followed by quenching of the crosslinking reaction with 1.25 M glycine. Cells were then harvested and incubated in a permeabilization buffer (0.25% Triton X-100) at room temperature for 30 minutes. Then, cells were washed with PBS, followed by incubation in the “click” (10 mM sodium ascorbate, 2 mM CuSO_4_, 10 mM biotin-azide, in PBS) or “no-click” (i.e., no biotin-azide) reaction cocktail for 2 hours at room temperature. After the reaction, cells were resuspended in lysis buffer (1% SDS, 50 mM Tris-HCl, pH 8.0) containing protease inhibitor (Roche), and cell lysates were sonicated 4 times using a Bioruptor at 4°C (30 seconds on and 30 seconds off) to generate 200~400 bp DNA fragments. After centrifugation, EdU-labeled DNA was immunoprecipitated from supernatants by incubation with streptavidin-MyOne T1 beads (Thermo Fisher Scientific; prewashed 3 times with PBS) for 4 hours at 4°C. The streptavidin-agarose beads containing the captured DNA-protein complexes were then centrifuged for 3 minutes at 1,800*g*. After washing for 5 minutes each with 1 mL cold lysis buffer, 1 mL of 1 M NaCl, and twice more with 1 mL lysis buffer, the beads were supplemented 1:1 (v/v of packed beads) with 2 × SDS-PAGE loading buffer and incubated at 95°C for 25 minutes to liberate the proteins. SDS-PAGE fractionation and immunoblotting were then performed.

### In situ protein interactions at nascent and stalled replication forks.

Cells were incubated with 125 μM EdU for 8 minutes, then EdU was removed and slides were washed 2 times with PBS before addition of media with hydroxyurea in the presence or absence of auranofin or NAC. Next, cells on glass coverslips were washed once with cold PBS and fixed with 4% paraformaldehyde for 5 minutes, then cells were permeabilized with 0.25% Triton X-100 in PBS for 15 minutes. Slides were washed in PBS twice for 5 minutes each. The freshly prepared click-reaction cocktail containing 2 mM CuSO_4_, 10 μM biotin-azide, and 100 mM sodium ascorbate was prepared in PBS and added to the slide for 1 hour incubation. Then, slides were blocked with 10% FBS in PBS for 1 hour at 37°C in a humidity chamber. Afterward, cells were incubated with the indicated primary antibodies at 4°C overnight (See supplemental materials for specific antibodies used.). A proximity ligation assay was performed according to the manufacturer’s instructions with Duolink In Situ Detection reagents (DUO92101, Sigma-Aldrich). Finally, coverslips were mounted on slides with Fluoroshield containing DAPI (Sigma-Aldrich). Images were captured with a Zeiss LSM 900 microscope using a 63× oil objective and quantified using ImageJ (NIH). RPA1 antibody specificity was validated by knockdown approaches.

### Mouse tumor models.

MDA-MB-231 cells (1.5 × 10^6^) and E0771 cells (3 × 10^5^) were resuspended with 200 μL PBS and orthotopically transplanted onto the mammary fat pad of 6-week-old NOD/SCID female mice and C57BL/6J female mice, respectively. Once the tumor volume had reached 40 mm^3^, mice were treated with rucaparib (20 mg/kg, 2% v/v DMSO, 10% v/v HPBCD, 2-hydroxylpropyl-β-cyclodextrin, in PBS) and/or auranofin (1, 5, or 25 mg/kg, 2% v/v DMSO, 10% v/v HPBCD, 2-hydroxylpropyl-β-cyclodextrin, in PBS) every 2 days in the absence or presence of NAC provided in the weekly updated drinking water (1 g/L). Similar strategies were used in drug administration for PyMT-driven mammary gland tumors in a C57BL/6J background (The Jackson Laboratory). Six to 10 animals per group were used in each type of mouse tumor model. Tumors were measured every 2 days using a vernier caliper, and the volume was calculated according to the formula 0.5 × length × width^2^.

### Statistics.

Data from biological triplicate experiments are presented as mean ± SD. An unpaired 2-tailed *t* test with Welch’s correction was used for comparing 2 groups of data. ANOVA with Bonferroni’s correction was used to compare multiple groups of data. One-way ANOVA followed by Tukey’s multiple-comparison test was used to analyze data with 1 independent variable, and 2-way ANOVA was used to analyze data with 2 independent variables. A *P* value of less than 0.01 was considered statistically significant. All statistical analyses used GraphPad Prism (version 10.1.2). Before statistical analysis, variation within each group of data and the assumptions of the tests were checked.

### Study approval.

All mouse studies were approved by the IACUC of Tianjin Medical University.

### Data availability.

All data generated or analyzed during this study are included in this published article and its supplemental materials. All data values supporting figures and analyses are included in the Supporting Data Values file. All unique reagents generated in this study are available from the corresponding authors with a completed materials transfer agreement. The mass spectrometry proteomics data have been deposited to the ProteomeXchange Consortium via the iProX partner repository (36) with the dataset identifier PXD066377.

## Author contributions

SM, YH, RG, XM, and LS conceived this project; SM, YH, RG, QC, QG, Y Yue, CC, Ling Liu, Z Yang, and YQ conducted experiments; SM, YH, RG, QC, QG, YY, CC, Ling Liu, Z Yang, and YQ acquired data; SM, YH, RG, QC, QG, Y Yue, CC, Ling Liu, Z Yang, YQ, XM, and LS analyzed data; Y Yang, KZ, FL, Lin Liu, NY, JH, JY, and Z Yao provided technical support and research resources; SM, YH, RG, XM, and LS wrote the manuscript.

## Funding support

This work is the result of NIH funding, in whole or in part, and is subject to the NIH Public Access Policy. Through acceptance of this federal funding, the NIH has been given a right to make the work publicly available in PubMed Central.

- National Key Research and Development Program of China (2024YFA1107402 to LS).
- National Natural Science Foundation of China (82425040 and 82230101 to LS, 81972791 and 12374413 to XM, 82372767 and 82003004 to SM, and 82303601 to CC).
- Tianjin Key Medical Discipline Construction Project (TJYXZDXK-3-003A).

## Supplementary Material

Supplemental data

Unedited blot and gel images

Supplemental table 1

Supporting data values

## Version 1 12/15/2025

Electronic publication
