# Supplementary material for: Auranofin attenuates TOPBP1-mediated ATR replication stress response and improves chemotherapeutic response in breast tumor models
Source: J Clin Invest. 2025 Dec 15;135(24):e180106. doi: 10.1172/JCI180106 (PMC12700547; doi:10.1172/JCI180106)
Supplement: Supplemental data [file jci-135-180106-s113.pdf]

Antibodies and reagents

The sources of antibodies against the following proteins or post-translational modifications were as follows: RPA1 (2198S, for IF), γH2AX (9718S, for IF), and phospho-CHK1 (Ser345) (2348S, for IB) from Cell Signaling Technology; BrdU (RPN202, for IF), PHF8 (HPA062015, for IF), 53BP1 (MAB3802, for IB), and FLAG (F3165, for IB and IP) from Sigma-Aldrich; RPA2 (ab2175, for IF), BLM (ab2179, for IB), Ki-67(ab16667, for IHC), and RPA1 (ab176467, for IB and IF) from Abcam; MDC1 (A300-052A, for IB), TOPBP1 (A300-111A, for IB, IF and IP), RAD9 (A300-890A, for IB), HTATSF1 (A302-023A, for IB), RPA2 pS4/8 (A300-245A, for IB and IF) and RPA2 pS33 (A300-246A, for IB and IF) from Bethyl Laboratories; b-actin (AC004, for IB), and RPA1 (A0990, for IF) from ABclonal; PHF8 (NB10093313, for IB) from Novus Biologicals; FANCJ (24436-1-AP, for IB, IF), CHK1 (25887-1-AP, for IB), PCNA (60097-1-Ig, for IB), PES1 (13553-1-AP, for IB), RACGAP1 (13739-1-AP, for IB), BRCA1 (22362-1-AP, for IB and IP), POLQ (28590-1-AP, for IB) and RPA2 (10412-1-AP, for IB) from Proteintech; PARP1 (sc-7150, for IB), TOPBP1 (sc-271043, for IF) from Santa Cruz Biotechnology; IdU/BrdU (347580, for IF) from BD; CldU/BrdU (MCA2060GA, for IF) from AbD Serotec; a-tubulin (YM3035, for IB), and H3 (YM3038, for IB) from Immunoway; Biotin (200-002-211, for IF) from Jackson ImmunoResearch. Protein G Magnetic Beads (10004D) were purchased from Invitrogen. CPT (C9911), HU (H8627), VE-821 (SML1415), IdU (I7125), CldU (C6891), blasticidin (15205), 1,6-Hexanediol (629-11-8), $KBrO_3$ (7758-01-2), and puromycin (P8833) were purchased from Sigma-Aldrich. Cisplatin (S1166), rucaparib

(S4948), auranofin (S4307), bortezomib (S1013), piperlongumine (S7551), mirin (S8096), N-acetylcysteine (1623), cyclosporine (S1514), and FDA-approved drug library (L1300-Z362685) were purchased from Selleck. PMX464 (HY-108534) was purchased from MCE. Human RPA complex was obtained from Enzymax, LLC (Catalog # 61).

Plasmids

The 3 × FLAG-tagged TOPBP1 was amplified from GFP-TOPBP1, a gift from Pro. Miiko Sokka (Department of Biology, University of Eastern Finland, Finland), and integrated into the pLenti-Puro vector. The GFP-tagged BRCT domain truncations of TOPBP1 carried by pLenti-NLS (nuclear localization signal)-GFP-3 × FLAG-Puro vector were generated by PCR-based standard cloning procedures. The FLAG-ECT2, FLAG-POLQ, and FLAG-GFP-RPA were amplified from cDNA from Open Biosystem, MAILGENE, and Origene, respectively, and cloned into pLenti-Puro vector. LgBit-BRCT 7-8-SmBit-PHF8/C and LgBit-BRCT 7-8-SmBit-FANCJ/C were chemically synthesized by General Bio and subcloned into the pLenti-Puro vector. The His-tagged BRCT 7-8 was carried by the pET-28a-smt vector, and the GST-tagged GFP-BRCT 6-8 was carried by the pGEX-6P-1 vector. mCherry-LacI was a gift from Dr. Mirek Dundr (Addgene plasmid # 18985). mCherry-LacI-TOPBP1 was a gift from Dr. Aziz Sancar (Addgene plasmid # 31313). TOPBP1 was cloned into a lentiviral backbone containing mCherry-Cry2 fusion protein with SV40 NLS (kindly provided by Dr. Jidong Zhu, Etern Biopharma, Shanghai, China) to generate opto-TOPBP1 constructs. F1491A of POLQ and F1411A, Y989A, F1071A, and

Y989A/F1071A of TOPBP1 mutations were generated by quick-change mutation strategy. pU6-tmpknot-GG-acceptor (Addgene plasmid # 174039) and pCMV-PEmax-P2A-hMLH1dn were gifts from Dr. David Liu (Harvard University). LentiGuide-Puro (Addgene plasmid # 52963) was a gift from Dr. Feng Zhang (Broad Institute).

Co-immunoprecipitation (Co-IP)

Cell lysates were prepared by incubating the cells in NETN buffer (50 mM Tris-HCl, pH 8.0, 150 mM NaCl, 0.2% Nonidet P-40, 2 mM EDTA) in the presence of protease inhibitor Cocktails (Roche) for 20 min at 4 ºC. This was followed by centrifugation at 14,000 g for 15 min at 4 ºC. For immunoprecipitation, about 500 μg of protein was incubated with control or specific antibodies (1-2 μg) for 12 hr at 4 ºC with constant rotation; 50 μl of 50% protein G magnetic beads (Invitrogen) was then added and the incubation was continued for an additional 2 hr. Beads were then washed five times using the lysis buffer. Between washes, the beads were collected by a magnetic stand (Invitrogen) at 4 ºC. The precipitated proteins were eluted from the beads by resuspending the beads in 2 × SDS-PAGE loading buffer and boiling for 10 min. The boiled immune complexes were subjected to SDS-PAGE followed by immunoblotting with appropriate antibodies.

Molecular docking

The compound auranofin served as the ligand for molecular docking in this study. All docking simulations were conducted using AutoDock 4.2. The crystal structure of TOPBP1

BRCT 7-8−APS was employed as the target conformation for docking (PDB: 7CMZ), with the docking grid centered on the geometric center of APS. The grid diameter was set to approximately 1.8 nm, and the grid size was established as 60 × 60 × 60 grids with a grid spacing of 0.375. The dimensions of the docking box were sufficiently large to encompass the potential binding activation pocket. The docking ligand was treated as conformationally flexible, and its torsional bonds were defined by AutoDock 4.2 based on chemical features. The final ligand docking pattern with the receptor was determined by selecting the decoy with the lowest binding free energy.

Protein expression and purification

A cDNA fragment encoding the BRCT 7-8 domain (amino acids 1264 to 1493) of TOPBP1 was cloned into a modified pET-28a-smt vector and the GFP-BRCT 6-8 (amino acids 884-1522) of TOPBP1 was cloned into a pGEX-6P-1 vector. The his-sumo-tagged or gst-tagged protein was expressed in the BL21 (DE3) strain of *Escherichia coli* by induction with 0.15 mM isopropyl-β-D-thiogalactopyranoside at 16 °C overnight. Cells were harvested by centrifugation and sonicated in buffer A [25 mM tris-HCl (pH 8.0), 500 mM NaCl, 10 mM imidazole, 1 mM EDTA, and 1 mM-β-mercaptoethanol (β-ME)]. Cell debris was removed by centrifugation at 4,000 g for 40 min at 4°C. The supernatant was loaded onto a Ni Sepharose Excel Column (GE Healthcare) followed by washing the beads with buffer A. The fusion protein was eluted with buffer B [25 mM tris-HCl (pH 8.0), 500 mM NaCl, 200 mM imidazole, 1 mM EDTA, and 1 mM β-ME]. His-tagged proteins were cleaved by sumo

protease to remove the sumo-tag, while GST-tagged proteins were cleaved by GST-tagged PreScission protease. The tag-free proteins were then loaded onto a HiTrap SP HP column (GE Healthcare) pre-equilibrated with buffer C [20 mM MES (pH 6.0), 100 mM NaCl, 1 mM EDTA, and 1 mM β-ME] and eluted with a linear gradient of 0.1 to 1.0 M NaCl. The eluted protein was concentrated by ultrafiltration and further purified by using a HiLoad Superdex 200 16/60 size-exclusion column (GE Healthcare) in a buffer containing 20 mM MES (pH 6.0), 300 mM NaCl, and 1 mM β-ME. High-purity fractions were pooled and concentrated to ~ 40 mg/ml and stored in PBS buffer for crystallization. Expression and purification procedures for mutant proteins were the same as those for the wild-type ones.

Lentiviral production

The lentivirus vectors encoding BRCT 7-8−PHF8/C and BRCT 7-8−FANCJ/C, and shRNAs against TrxR and TOPBP1were individually co-transfected with three assistant vectors – pMDLg/pRRE, pRSV-REV, and pVSV-G into HEK 293T cells. Viral supernatants were collected 48 hr later, clarified by filtration, and concentrated by ultracentrifugation. The lentivirus was then used to transduce cells, followed by antibiotic selection to generate stable lines.

Gel shift assay

DNA substrates labelled with 5' biotin-labelled ssDNA (GCTTGCATGCCTGCAGGCCAGCCTCAATCTCATC, 10 nM) were incubated with

indicated amounts of proteins at room temperature in 1× binding buffer (25 mM Tris, pH 7.5, 200 mM NaCl, 5 mM $MgCl_2$, 1 mM DTT, 5% glycerol, and 0.05% Triton X-100) for 20 min. The reaction mixture (20 μL in total) was then loaded with 2 μL 10 × loading dye and resolved in 4% native acrylamide/Bis gel in cold 0.5 × TBE buffer (44.5 mM Tris, 44.5 mM boric acid, and 0.5 mM EDTA, pH 8.3). After electrophoresis, the gels were transferred to a nylon membrane. A LightShift™ Chemiluminescent EMSA Kit (Thermo Scientific) was used to detect DNA-protein interactions. Signals were detected with a Tanon-5200 imager (Tanon).

Subcellular fractionation

Briefly, cells were harvested at the indicated time after treatment with 4 mM HU and aranofin in the presence or absence of NAC and subsequently lysed for 15 min with 10 volumes of low-salt NTEN buffer (20 mM Tris-HCl, pH 8.0, 10 mM NaCl, 1.5 mM $MgCl_2$, 1 mM EDTA, 0.5% Nonidet P-40, 20 mM NaF, 1 mM Na_3VO_4, 1 mg/ml aprotinin, and 1 mg/ml pepstatin). The chromatin-enriched pellet was washed three times with cold PBS. The insoluble chromatin fractions were resuspended in 0.2 M HCl for 30 min on ice. The resulting soluble extraction was then neutralized with 1 M Tris-HCl, pH 8.5 for further analysis.

Procedure for preparation of biotin-auranofin

The synthesis of biotin-auranofin was performed through a multi-step route by WuXi

AppTec. The process began with the reaction of a tetraacetylated thiopyran derivative with a trityl-protecting group in pyridine, followed by deacetylation using sodium methoxide. Subsequent steps included silyl protection of the hydroxyl group, reacetylation, and biotin conjugation via DCC/DMAP coupling. The trityl group was then removed under acidic conditions, and the free thiol intermediate was reacted with chloroauric acid and triethylphosphine to form the gold(I) complex. After LCMS confirmation, the crude product was purified by silica gel chromatography and preparative HPLC, yielding the final compound as a white solid (30 mg, 16.6% yield, 85.4% purity).

Mass spectrometry analysis and data processing

The streptavidin magnetic beads with captured proteins were suspended in 50 mM NH_4HCO_3 and treated with dithiothreitol (5 mM) at 56 °C for 1h, followed by alkylation with iodoacetamide (15 mM) at room temperature for 45 min in the dark, and the unreacted iodoacetamide was neutralized with 30 mM cysteine. The proteins were incubated with trypsin (Promega) overnight at 37 °C. Tryptic peptides in the supernatant were dried in a SpeedVac, and desalted using a μ-C18 Ziptip (Millipore). After desalting, the digested sample was injected into a Nano-LC system (EASY-nLC 1200, Thermo Fisher Scientific). Each sample was separated by a C18 column (75 μm inner-diameter×25 cm, 3 μm) at a flow rate of 300 nL/min. The HPLC gradient was as follows: 5% to 7% solvent B (0.1% formic acid in 80% acetonitrile) in 2 min, 7% to 26% solvent B in 60 min, 26% to 38% solvent B in 20 min, 38% to 100% solvent B in 2 min and hold for 6 min at 100% solvent

B. The HPLC elute was electrosprayed directly into an Orbitrap Eclipse mass spectrometer (Thermo Fisher Scientific). The source was operated at 2.2 kV. The mass spectrometric analysis was carried out in a data-dependent mode. For MS1 survey scan, automatic gain control (AGC) target is 4e5 and the resolution is 60,000. The MS2 spectra were acquired with 15000 resolution. MS/MS data were searched using MaxQuant (Version 1.5.5). Peptide sequences were searched using trypsin specificity and allowing a maximum of two missed cleavages. Carbamidomethylation on cysteine was specified as fixed modification. Oxidation of methionine and acetylation on N-terminal and lysine were set as variable modifications. A mass tolerance of 10 ppm was used for precursor ions and a tolerance of 20 ppm was used for fragment ions. Regarding this all-peptide quantification approach by MaxQuant, protein intensity is the sum of all identified peptide intensities, peptide-feature intensities are taken at the peak maximum over the elution profile and include all isotopic peaks. A fold change > 2 and a p-value < 0.01 were set to screen the significantly different proteins; proteins with a unique peptide count < 2 were excluded. Meanwhile, proteins were quantified via Proteome Discoverer 3.0 using the Top-3 method. Similar to MaxQuant, a trypsin-specific search was chosen, and a maximum of two missed cleavages was allowed. Dynamic modifications included oxidation at methionine and acetylation at N-terminal. Fixed modification of carbamidomethyl at cysteine was chosen. The MS and MS/MS results were searched with a peptide ion mass tolerance of 10 ppm and a fragment ion mass tolerance of 0.02 Da. When using the Top-3 method, protein intensity is calculated as the average of the three most abundant distinct peptides, and fold change > 4

and p-value < 0.01 were set to screen the significantly different proteins. A total of 3 technical replicates in each treatment were analyzed by the all-peptide-based and Top-3-based label-free quantification methods. The mass spectrometry proteomics data have been deposited to the ProteomeXchange Consortium via the iProX partner repository with the dataset identifier PXD066377.

Cell survival assay

Cells were plated into 96-well plates at a density of 2000 cells/well. After 24 hr, cells were treated with various doses of genotoxic agents for 72 hr. Then, Cell Titer Aqueous One Solution (Promega) was added to each well according to the manufacturer's instructions and cell survival was determined after 1 hr incubation by measuring the absorbance at 490 nm using a Bio-Rad plate-reader (model 550; Bio-Rad).

Immunohistochemistry

Immunohistochemistry (IHC) was performed on paraffin-embedded tissue sections. Briefly, sections were baked at 65°C for 1 hour, deparaffinized in xylene, and rehydrated through a graded ethanol series. Endogenous peroxidase activity was quenched by incubation in 3% (v/v) hydrogen peroxide in methanol for 30 minutes, followed by three 3-minute washes with phosphate-buffered saline (PBS). Antigen retrieval was performed by high-pressure heating in 0.01 M citrate buffer (pH 6.0) for 2 minutes, after which sections were cooled to room temperature. After washing with PBS, sections were blocked with 10% (v/v)

normal goat serum for 30 minutes and incubated overnight at 4°C with the primary antibody diluted in PBS containing 10% normal goat serum. Following three 5-minute PBS washes, sections were incubated with a peroxidase-conjugated secondary antibody (ZSGB-BIO, Cat#: PV-9000) for 1 hour at room temperature and washed three times with PBS. Finally, sections were developed using DAB solution (ZSGB-BIO, Cat#: ZLI-9018) and counterstained with hematoxylin according to the manufacturer's protocol.

TUNEL

For the TUNEL assay, a Colorimetric TUNEL Apoptosis Assay Kit (Cat#: C1098; Beyotime) was used. Briefly, tissue sections were rehydrated and washed with distilled water prior to staining. Following the manufacturer’s instructions, the tissue sections were labeled with biotin and stained using DAB. The nuclei were then counterstained with hematoxylin.

Supplemental Figure 1 (Figure 1 continued). Zinc pyrithione and sanguinarine chloride could not compete with PHF8/APS and pFANCJ peptides for TOPBP1 BRCT 7-8 binding. (**A**) BLI analysis of the inhibitory effect of Zinc pyrithione (left) and sanguinarine chloride (right) on BRCT 7-8 binding to PHF8/APS peptide. His-tagged BRCT 7-8 (1 μM) was preincubated with these drugs at the indicated concentrations before examining peptide-protein interaction. BLI sensorgrams are shown. Black lines, fitted curves; color traces, raw data. (**B**) BLI analysis of the inhibitory effect of Zinc pyrithione on BRCT 7-8 binding to phosphorylated Thr1133-containing peptide of FANCJ (pFANCJ). His-tagged BRCT 7-8 (1 μM) was preincubated with Zinc pyrithione at the indicated concentrations before examining peptide-protein interaction. BLI sensorgrams are shown. Black lines, fitted curves; color traces, raw data.

Supplemental Figure 2 (Figure 2 continued). Auranofin disrupts the interaction of TOPBP1 with PHF8 and FANCJ. (**A**) Co-IP analysis of the interactions of TOPBP1-PHF8 and TOPBP1-FANCJ in HeLa or U2OS cells treated by auranofin (2 μM, 2 hr) and cyclosporine (10 μM, 4 hr). (**B**) Examination of His-tagged recombinant proteins purified from bacterial cells by coomassie brilliant blue (CBB) staining. The molecular weight is shown. (**C**) Co-IP analysis of the interaction of FLAG-tagged TOPBP1 BRCT 7-8 with POLQ with cellular extracts from HeLa cells treated with auranofin (2 μM, 2 hr). (**D**) Immunostaining and confocal microscopy analysis of mitotic TOPBP1 and FLAG-POLQ foci formation in U2OS cells under auranofin treatment (2 μM, 2 hr). Nocodazole-

synchronized cells (12 hr, 100 ng/mL) were irradiated (1 Gy) and collected 4 hr later. The foci number of POLQ and TOPBP1 in mitotic cells was quantified and shown (n > 29). TOPBP1 loading onto mitotic DSBs requires BRCT 0-2 (resistant to auranofin), while POLQ recruitment to these break sites depends on its interaction with BRCT 7-8 (sensitive to auranofin). (**E**) A comparison between the co-crystal structure of PHF8 (amino acid from 842 to 863) and BRCT 7-8 (PDB: 7CMZ), co-crystal structure of FANCJ (amino acid from 1128 to 1142) and BRCT 7-8 (PDB: 3AL3), and the alphaFold-multimer-predicted structure of POLQ (amino acid from 1480 to 1498) and BRCT 7-8. A superimposed model is shown. The POLQ, PHF8, and FANCJ peptides are displayed in green, purple, and yellow, respectively. Dashed lines indicate π-π interaction and distances are labeled and expressed in angstroms. In the upper panel, BRCT 7-8 is displayed as a surface colored according to its electrostatic potential (red, negatively charged; blue, positively charged), and the lower panel is shown in a cartoon model. (**F**) Co-IP analysis of the interaction of FLAG-TOPBP1/Wt or FLAG-TOPBP1/F1411A with POLQ in nocodazole-synchronized HeLa cells (12 hr, 100 ng/mL). (**G**) Co-IP analysis of the interaction of FLAG-POLQ/Wt or FLAG-POLQ/F1491A with TOPBP1 in nocodazole-synchronized HeLa cells (12 hr, 100 ng/mL). (**H**) Immunostaining and confocal microscopy analysis of mitotic FLAG-POLQ/Wt or FLAG-POLQ/F1491A foci formation in U2OS cells. Nocodazole-synchronized cells (12 hr, 100 ng/mL) were irradiated (1 Gy) and collected 4 hr later. The foci number in mitotic cells was quantified and shown (n > 26). (**I**) Co-IP analysis of the interactions of FLAG-TOPBP1 BRCT 0-2

and FLAG-TOPBP1 BRCT 4-5 with different interaction partners as indicated using cellular extracts from HeLa cells under auranofin treatment (2 μM, 2 hr). (**J**) Co-IP analysis of the interaction between BRCA1 and FANCJ, and the interaction between FLAG-ECT2 and RACGAP1 with cellular extracts from HeLa cells under auranofin treatment (2 μM, 2 hr). (**K**) Quantitative mass spectrometry analysis of biotin-auranofin interaction proteins with cellular extracts from HeLa cells (n = 3). Proteins were quantified using MaxQuant with a false discovery rate (FDR) < 1% and at least two unique peptides, and the relative ratio indicates the biotin-auranofin group to the biotin-linker group. The chemical structure of biotin-tagged auranofin is shown. (**L**) Pull-down analysis of the interaction between biotin-auranofin and BRCT domain-containing proteins with cellular extracts from HeLa cells. PARP1 and PES1 are potential interactors of auranofin retrieved from mass spectrometry data. (**M**) The ratio of proteome from the same dataset as in (**K**) was re-quantified using Proteome Discoverer via the Top-3 intensity methods. (**N**) TOPBP1 abundance comparison between groups is quantified by all unique peptide intensities and Top-3 intensity methods. Data are mean ± SDs for (**D**) and (**H**) from biological triplicate experiments. ***P* < 0.01; NS, not significant; unpaired t-test with Welch's correction for (**D**), (**H**), and (**N**); Scale bars, 10 μm.

Supplemental Figure 3 (Figure 3 continued). Auranofin impairs TOPBP1 recruitment and ATR activation. (**A**) Evaluation of the antioxidant activity of NAC by immunostaining and confocal microscopy. HeLa cells were treated with $KBrO_3$ (40 mM)

in the absence or presence of NAC (50 μM) for 0.5 hr followed by fixation. The intensity of 8-oxoG, a ROS-activating marker, was quantified and shown (n > 50). Data are mean ± SDs from biological triplicate experiments. $^{**}P < 0.01$; one-way ANOVA followed by Tukey's multiple comparisons test. Scale bars, 10 μm. (**B**) Immunostaining and confocal microscopy analysis of TOPBP1 intensity. Cells treated with DMSO or auranofin (2 μM, 2 hr) followed by EdU-labelling for 1 additional hour. These cells were then fixed without pre-extraction or pre-treated with 0.5% Triton X-100 for 5 min on ice to extract non-chromatin fractions before fixation. The intensity of TOPBP1 in EdU-positive cells was quantified and shown (n > 65). Data are mean ± SDs from biological triplicate experiments. NS, not significant; one-way ANOVA followed by Tukey's multiple comparisons test. Scale bars, 10 μm.

Supplemental Figure 4 (Figure 4 continued). Auranofin dissolves TOPBP1 liquid-liquid condensate. (**A**) Conserved aromatic residues among TOPBP1, PHF8, and FANCJ are highlighted in orange, and a schematic diagram of TOPBP1 BRCT 6-8 domain is shown. AAD, ATR activation domain. (**B**) Model of BRCT 7-8 in complex with Y989- or F1071-containing peptides, as predicted by alphaFold-multimer. The Y989- and F1071-containing peptides are displayed in purple and orange, respectively. Dashed lines indicate π-π interaction and distances are labeled and expressed in angstroms. In the left panel, BRCT 7-8 is displayed as a surface colored according to its electrostatic potential (red, negatively charged; blue, positively charged), and the right panel is shown in a

cartoon model. (**C**) BLI analysis of BRCT 7-8 binding to biotin-conjugated Y989/Wt and Y989A peptides. BLI sensorgrams for each group are shown. Color traces represent fitted curves. (**D**) BLI analysis of BRCT 7-8 binding to biotin-conjugated F1071/Wt and F1071A peptides. BLI sensorgrams for each group are shown. Color traces represent fitted curves. (**E**) BLI analysis of the inhibitory effect of auranofin on BRCT 7-8 binding to TOPBP1/Y989 peptide and TOPBP1/F1071 peptide. His-tagged BRCT 7-8 (1 μM) was preincubated with auranofin at the indicated concentrations before examining peptide-protein interactions. The BLI sensorgrams are shown. Color traces represent fitted curves. (**F**) Liquid droplet formation of BRCT 6-8/Wt, Y989A, F1071A, and Y989A/F1071A (10 μM) observed by confocal microscopy in PEG8000 solution. The occupied areas of the droplets were quantified and shown. The GST-GFP-tagged recombinant proteins purified from bacterial cells were examined by CBB staining. (**G**) Representative micrographs and quantitative analysis of time-lapse opto-droplet formation of mCherry-Cry2-tagged proteins as indicated after blue light activation ($n > 15$). (**H**) Representative micrographs and quantitative analysis of the puncta intensity of PHF8 and mCherry-LacI-TOPBP1 in the presence of vehicle, auranofin (2 μM, 2 hr), and 1,6-hexanediol (1,6-HD, 2.5% for 5 min) as indicated. The occupied area of PHF8 and mCherry-marked puncta in U2OS cells stably integrated with 256 × LacO repeats was quantified and normalized to that of the corresponding nucleus ($n > 20$). (**I**) Representative micrographs and quantitative analysis of the puncta intensity of FANCJ and mCherry-LacI-TOPBP1 in the presence of vehicle, auranofin (2 μM, 2 hr), and 1,6-

hexanediol (1,6-HD, 2.5% for 5 min) as indicated. The occupied area of FANCJ and mCherry-marked puncta in U2OS cells stably integrated with 256 × LacO repeats was quantified and normalized to that of the corresponding nucleus (n > 17). (**J**) Immunoblotting analysis of the expression of the indicated proteins with cellular extracts from (**I**). (**K**) Representative micrographs and quantitative analysis of the puncta intensity of mCherry-LacI-TOPBP1 and mCherry-LacI in U2OS cells expressing the indicated siRNAs. The occupied area of mCherry-marked puncta was quantified and normalized to that of the corresponding nucleus (n ⩾ 15). (**L**) Immunoblotting analysis of the knockdown effects using cellular extracts from (**K**).

Data are mean ± SDs for (**F**-**I**) and (**K**) from biological triplicate experiments. $^{**}P < 0.01$; NS, not significant; one-way ANOVA followed by Tukey's multiple comparisons test for (**F**), (**H**-**I**), and (**K**); two-way ANOVA for (**G**). Scale bars, 10 μm.

Supplemental Figure 5 (Figure 5 continued). Auranofin prevents RPA loading to perturbed replication forks. (**A**) Pull-down analysis of the interaction between biotin-auranofin and the indicated proteins. (**B**) Co-IP analysis of the interaction of FLAG-GFP-tagged RPA1 or RPA2 with FANCJ using cellular extracts from HeLa cells treated by auranofin (2 μM, 2 hr). (**C**-**D**) Immunostaining and confocal microscopy analysis of TOPBP1 (**C**), RPA2 (**D**), and RPA2 pS33 (**D**) foci formation. Cells were transfected with siRNAs as indicated or treated with bortezomib (10 μM) for 4 hr before collection. Replication stress was induced by CPT (2 μM) for 1 hr, and the actively replicating cells

were labeled by EdU (10 μM) for 1 hr. Cells were then pre-extracted and fixed. The intensity of foci in EdU-positive cells was quantified and shown (n > 55). (**E**) Examination of the siRNA-mediated knockdown effect of UCHL5 and USP14 by reverse transcription coupled quantitative PCR. (**F**) Analysis of ssDNA-RPA binding in the absence or presence of auranofin. Gel shift assays were performed with 5' biotin-labeled 34-nt ssDNA (10 nM) and an increasing amount of RPA in the absence or presence of different doses of auranofin followed by electrophoresis and visualization. Free and bound ssDNA is marked as indicated. (**G**) Immunostaining and confocal microscopy analysis of BrdU foci formation in auranofin-treated U2OS cells (2 μM, 2 hr). Replication stress was induced by HU (1 mM) for 4 hr, and the actively replicating cells were labeled by EdU (10 μM) for 1 hr before collection. Cells were then pre-extracted and fixed. The intensity of foci in EdU-positive cells was quantified and shown (n > 46). Data are mean ± SDs for (**C-E**) and (**G**) from biological triplicate experiments. $^{**}P < 0.01$; NS, not significant; one-way ANOVA followed by Tukey's multiple comparisons test for (**C-E**) and (**G**).

Supplemental Figure 6 (Figure 6 continued). Survival analysis of the synthetic lethality of auranofin and CPT (A) or rucaparib (B) in HMECs. Auranofin at a concentration of 1.5 μM was used. Data are mean ± SDs from one representative data of biological triplicate experiments. NS, not significant; two-way ANOVA.

Supplemental Figure 7 (Figure 7 continued). Breast tumor is synthetically susceptible to the action of auranofin and rucaparib. (**A**) Mouse weight of mice bearing xenografts from MDA-MB-231 cells. NOD/SCID mice carrying tumors were treated with rucaparib, auranofin, or both every two days. (**B**) Mouse weight of mice bearing xenografts from E0771 cells. C57BL/6J mice carrying tumors were treated with rucaparib, auranofin, or both every two days. (**C**) Mouse weight of mice bearing genetically engineered mammary gland tumors. PyMT mice carrying tumors were treated with rucaparib, auranofin, or both every two days. (**D**-**F**) Mouse weight of mice bearing tumors of MDA-MB-231 cells (**D**), E0771 cells (**E**), and genetically engineered mammary gland tumors (**F**) under combinatorial auranofin and rucaparib treatment in the absence or presence of NAC. (**G**) Tumor size and weight of xenografts from E0771 cells. C57BL/6J mice carrying tumors were treated with vehicle, different doses of auranofin (1, 5, 25 mg/kg), or both auranofin (5 mg/kg) and rucaparib (25 mg/kg) every two days. (**H**) Mouse weight of mice bearing tumor xenografts from (**G**). (**I**) Ki-67, HE, and TUNEL staining of intestinal tissues using mice from (**G**). Representative images from 3 mice in each treatment are shown. Scale bars, 100 μm. (**J**) Quantification of key complete blood count (CBC) parameters of C57BL/6J mice used in (**G**). RBC, red blood cell count; WBC, white blood cell count; MCHC, mean corpuscular hemoglobin concentration; PLT, platelet count; MCV, mean corpuscular volume; MCH, mean corpuscular hemoglobin; HCT, hematocrit; HGB, hemoglobin; MPV, mean platelet volume; RDW-SD, red cell distribution width-standard deviation; RDW-CV, red cell distribution width-coefficient of

variation.

Data are mean ± SDs for (**A**-**H**) and (**J**). ***$P < 0.01$; NS, not significant; two-way ANOVA for (**A**-**F**) and (**H**), one-way ANOVA followed by Tukey's multiple comparisons test for (**G**) and (**J**).

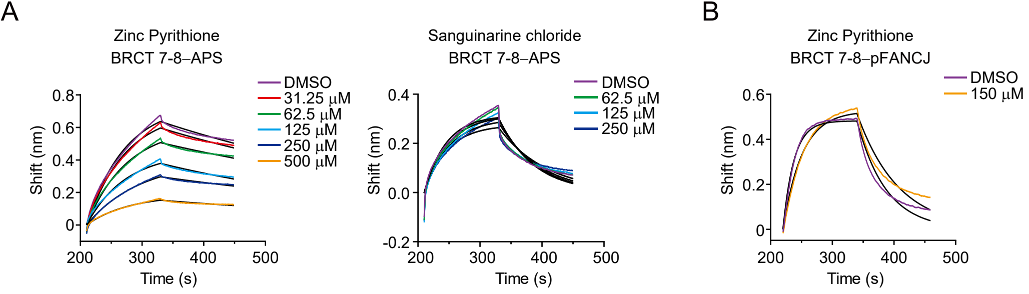

Supplemental Figure 1 (Figure 1 continued). Zinc pyrithione and sanguinarine chloride could not compete with PHF8/APS and pFANCJ peptides for TOPBP1 BRCT 7-8 binding. (**A**) BLI analysis of the inhibitory effect of Zinc pyrithione (left) and sanguinarine chloride (right) on BRCT 7-8 binding to PHF8/APS peptide. His-tagged BRCT 7-8 (1 μM) was preincubated with these drugs at the indicated concentrations before examining peptide-protein interaction. BLI sensorgrams are shown. Black lines, fitted curves; color traces, raw data. (**B**) BLI analysis of the inhibitory effect of Zinc pyrithione on BRCT 7-8 binding to phosphorylated Thr1133-containing peptide of FANCJ (pFANCJ). His-tagged BRCT 7-8 (1 μM) was preincubated with Zinc pyrithione at the indicated concentrations before examining peptide-protein interaction. BLI sensorgrams are shown. Black lines, fitted curves; color traces, raw data.

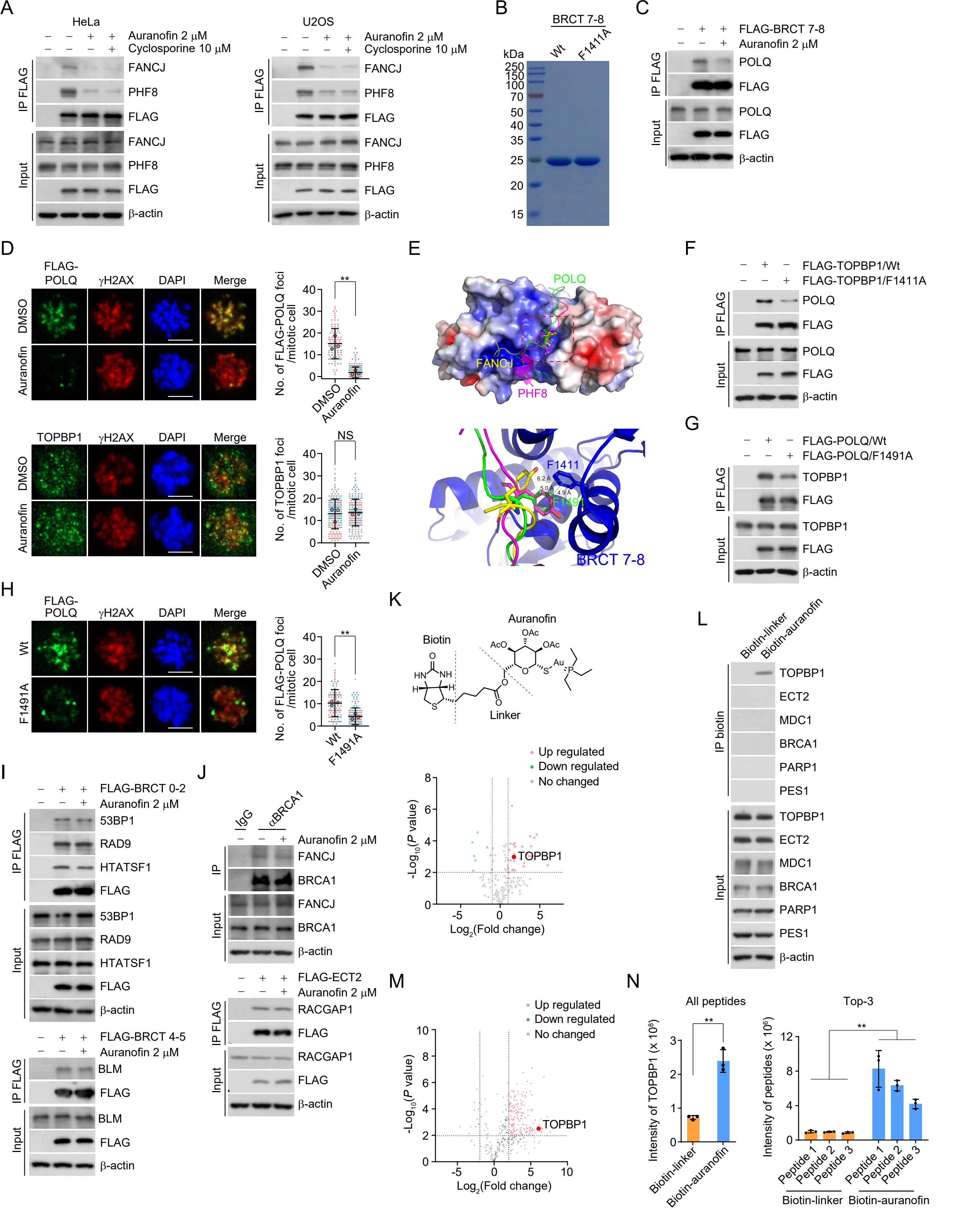

A
HeLa
Auranofin 2 μM
Cyclosporine 10 μM
IP FLAG
FANCJ
PHF8
FLAG
Input
FANCJ
PHF8
FLAG
β-actin
U2OS
Auranofin 2 μM
Cyclosporine 10 μM
B
BRCT 7-8
Wt
F1411A
kDa
250
150
100
70
50
40
35
25
20
15
C
FLAG-BRCT 7-8
Auranofin 2 μM
POLQ
FLAG
β-actin
D
FLAG-POLQ
γH2AX
DAPI
Merge
DMSO
Auranofin
No. of FLAG-POLQ foci /mitotic cell
TOPBP1
No. of TOPBP1 foci /mitotic cell
NS
E
POLQ
FANCJ
PHF8
F1411
F1491
BRCT 7-8
F
FLAG-TOPBP1/Wt
FLAG-TOPBP1/F1411A
G
FLAG-POLQ/Wt
FLAG-POLQ/F1491A
TOPBP1
H
Wt
F1491A
I
FLAG-BRCT 0-2
53BP1
RAD9
HTATSF1
FLAG-BRCT 4-5
BLM
J
IgG
αBRCA1
IP
BRCA1
FLAG-ECT2
RACGAP1
K
Auranofin
Biotin
Linker
Up regulated
Down regulated
No changed
-Log10(P value)
Log2(Fold change)
L
Biotin-linker
Biotin-auranofin
IP biotin
ECT2
MDC1
PARP1
PES1
M
N
All peptides
Top-3
Intensity of TOPBP1 (x 10^8)
Intensity of peptides (x 10^6)
Peptide 1
Peptide 2
Peptide 3

Supplemental Figure 2 (Figure 2 continued). Auranofin disrupts the interaction of TOPBP1 with PHF8 and FANCJ. (**A**) Co-IP analysis of the interactions of TOPBP1-PHF8 and TOPBP1-FANCJ in HeLa or U2OS cells treated by auranofin (2 μM, 2 hr) and cyclosporine (10 μM, 4 hr). (**B**) Examination of His-tagged recombinant proteins purified from bacterial cells by coomassie brilliant blue (CBB) staining. The molecular weight is shown. (**C**) Co-IP analysis of the interaction of FLAG-tagged TOPBP1 BRCT 7-8 with POLQ with cellular extracts from HeLa cells treated with auranofin (2 μM, 2 hr). (**D**) Immunostaining and confocal microscopy analysis of mitotic TOPBP1 and FLAG-POLQ foci formation in U2OS cells under auranofin treatment (2 μM, 2 hr). Nocodazole-synchronized cells (12 hr, 100 ng/mL) were irradiated (1 Gy) and collected 4 hr later. The foci number of POLQ and TOPBP1 in mitotic cells was quantified and shown (n > 29). TOPBP1 loading onto mitotic DSBs requires BRCT 0-2 (resistant to auranofin), while POLQ recruitment to these break sites depends on its interaction with BRCT 7-8 (sensitive to auranofin). (**E**) A comparison between the co-crystal structure of PHF8 (amino acid from 842 to 863) and BRCT 7-8 (PDB: 7CMZ), co-crystal structure of FANCJ (amino acid from 1128 to 1142) and BRCT 7-8 (PDB: 3AL3), and the alphaFold-multimer-predicted structure of POLQ (amino acid from 1480 to 1498) and BRCT 7-8. A superimposed model is shown. The POLQ, PHF8, and FANCJ peptides are displayed in green, purple, and yellow, respectively. Dashed lines indicate π-π interaction and distances are labeled and expressed in angstroms. In the upper panel, BRCT 7-8 is displayed as a surface colored according to its electrostatic potential (red, negatively charged; blue, positively charged), and the lower panel is shown in a cartoon model. (**F**) Co-IP analysis of the interaction of FLAG-TOPBP1/Wt or FLAG-TOPBP1/F1411A with POLQ in nocodazole-synchronized HeLa cells (12 hr, 100 ng/mL). (**G**) Co-IP analysis of the interaction of FLAG-POLQ/Wt or FLAG-POLQ/F1491A with TOPBP1 in nocodazole-synchronized HeLa cells (12 hr, 100 ng/mL). (**H**) Immunostaining and confocal microscopy analysis of mitotic FLAG-POLQ/Wt or FLAG-POLQ/F1491A foci formation in U2OS cells. Nocodazole-synchronized cells (12 hr, 100 ng/mL) were irradiated (1 Gy) and collected 4 hr later. The foci number in mitotic cells was quantified and shown (n > 26). (**I**) Co-IP analysis of the interactions of FLAG-TOPBP1 BRCT 0-2 and FLAG-TOPBP1 BRCT 4-5 with different interaction partners as indicated using cellular extracts from HeLa cells under auranofin treatment (2 μM, 2 hr). (**J**) Co-IP analysis of the interaction between BRCA1 and FANCJ, and the interaction between FLAG-ECT2 and RACGAP1 with cellular extracts from HeLa cells under auranofin treatment (2 μM, 2 hr). (**K**) Quantitative mass spectrometry analysis of biotin-auranofin interaction proteins with cellular extracts from HeLa cells (n = 3). Proteins were quantified using MaxQuant with a false discovery rate (FDR) < 1% and at least two unique peptides, and the relative ratio indicates the biotin-auranofin group to the biotin-linker group. The chemical structure of biotin-tagged auranofin is shown. (**L**) Pull-down analysis of the interaction between biotin-auranofin and BRCT domain-containing proteins with cellular extracts from HeLa cells. PARP1 and PES1 are potential interactors of auranofin retrieved from mass spectrometry data. (**M**) The ratio of proteome from the same dataset as in (**K**) was re-quantified using Proteome Discoverer via the Top-3 intensity methods. (**N**) TOPBP1 abundance comparison between groups is quantified by all unique peptide intensities and Top-3 intensity methods. Data are mean ± SDs for (**D**) and (**H**) from biological triplicate experiments. $^{**}P < 0.01$; NS, not significant; unpaired t-test with Welch's correction for (**D**), (**H**), and (**N**); Scale bars, 10 μm.

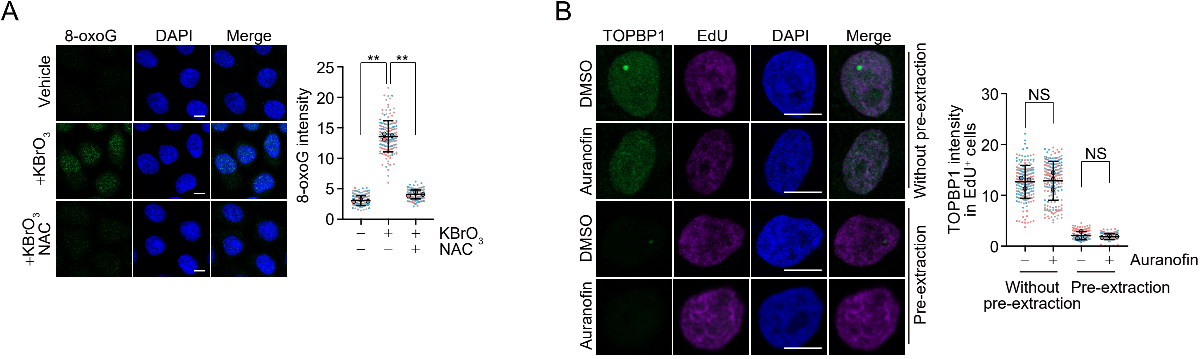

Supplemental Figure 3 (Figure 3 continued). Auranofin impairs TOPBP1 recruitment and ATR activation. (**A**) Evaluation of the antioxidant activity of NAC by immunostaining and confocal microscopy. HeLa cells were treated with KBrO3 (40 mM) in the absence or presence of NAC (50 μM) for 0.5 hr followed by fixation. The intensity of 8-oxoG, a ROS-activating marker, was quantified and shown (n > 50). Data are mean ± SDs from biological triplicate experiments. ***P* < 0.01; one-way ANOVA followed by Tukey's multiple comparisons test. Scale bars, 10 μm. (**B**) Immunostaining and confocal microscopy analysis of TOPBP1 intensity. Cells treated with DMSO or auranofin (2 μM, 2 hr) followed by EdU-labelling for 1 additional hour. These cells were then fixed without pre-extraction or pre-treated with 0.5% Triton X-100 for 5 min on ice to extract non-chromatin fractions before fixation. The intensity of TOPBP1 in EdU-positive cells was quantified and shown (n > 65). Data are mean ± SDs from biological triplicate experiments. NS, not significant; one-way ANOVA followed by Tukey's multiple comparisons test. Scale bars, 10 μm.

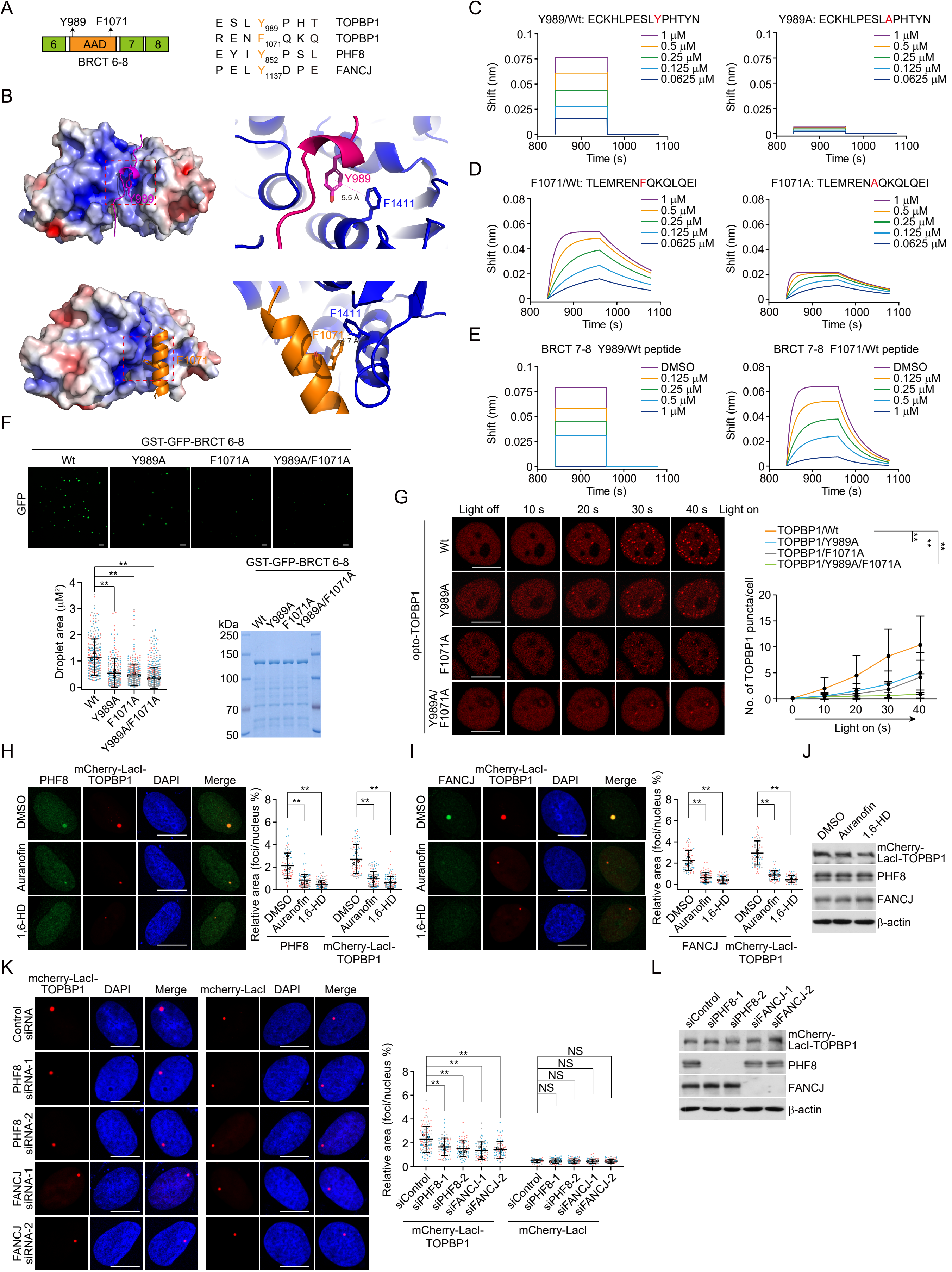
A
Y989 F1071
6 AAD 7 8
BRCT 6-8
E S L Y989 P H T TOPBP1
R E N F1071 Q K Q TOPBP1
E Y I Y852 P S L PHF8
P E L Y1137 D P E FANCJ
B
Y989
5.5 Å
F1411
F1071
4.7 Å
C
Y989/Wt: ECKHLPESLYPHTYN
Y989A: ECKHLPESLAPHTYN
D
F1071/Wt: TLEMRENFQKQLQEI
F1071A: TLEMRENAQKQLQEI
E
BRCT 7-8–Y989/Wt peptide
BRCT 7-8–F1071/Wt peptide
1 μM
0.5 μM
0.25 μM
0.125 μM
0.0625 μM
DMSO
Shift (nm)
Time (s)
F
GST-GFP-BRCT 6-8
Wt Y989A F1071A Y989A/F1071A
GFP
Droplet area (μM²)
kDa
G
Light off 10 s 20 s 30 s 40 s Light on
opto-TOPBP1
TOPBP1/Wt
TOPBP1/Y989A
TOPBP1/F1071A
TOPBP1/Y989A/F1071A
No. of TOPBP1 puncta/cell
Light on (s)
H
PHF8 mCherry-LacI-TOPBP1 DAPI Merge
DMSO
Auranofin
1,6-HD
Relative area (foci/nucleus %)
I
FANCJ mCherry-LacI-TOPBP1 DAPI Merge
J
mCherry-LacI-TOPBP1
PHF8
FANCJ
β-actin
K
mcherry-LacI-TOPBP1 DAPI Merge mcherry-LacI DAPI Merge
Control siRNA
PHF8 siRNA-1
PHF8 siRNA-2
FANCJ siRNA-1
FANCJ siRNA-2
siControl siPHF8-1 siPHF8-2 siFANCJ-1 siFANCJ-2
mCherry-LacI-TOPBP1
mCherry-LacI
NS
L
siControl siPHF8-1 siPHF8-2 siFANCJ-1 siFANCJ-2

Supplemental Figure 4 (Figure 4 continued). Auranofin dissolves TOPBP1 liquid-liquid condensate. (**A**) Conserved aromatic residues among TOPBP1, PHF8, and FANCJ are highlighted in orange, and a schematic diagram of TOPBP1 BRCT 6-8 domain is shown. AAD, ATR activation domain. (**B**) Model of BRCT 7-8 in complex with Y989- or F1071-containing peptides, as predicted by alphaFold-multimer. The Y989- and F1071-containing peptides are displayed in purple and orange, respectively. Dashed lines indicate π-π interaction and distances are labeled and expressed in angstroms. In the left panel, BRCT 7-8 is displayed as a surface colored according to its electrostatic potential (red, negatively charged; blue, positively charged), and the right panel is shown in a cartoon model. (**C**) BLI analysis of BRCT 7-8 binding to biotin-conjugated Y989/Wt and Y989A peptides. BLI sensorgrams for each group are shown. Color traces represent fitted curves. (**D**) BLI analysis of BRCT 7-8 binding to biotin-conjugated F1071/Wt and F1071A peptides. BLI sensorgrams for each group are shown. Color traces represent fitted curves. (**E**) BLI analysis of the inhibitory effect of auranofin on BRCT 7-8 binding to TOPBP1/Y989 peptide and TOPBP1/F1071 peptide. His-tagged BRCT 7-8 (1 μM) was preincubated with auranofin at the indicated concentrations before examining peptide-protein interactions. The BLI sensorgrams are shown. Color traces represent fitted curves. (**F**) Liquid droplet formation of BRCT 6-8/Wt, Y989A, F1071A, and Y989A/F1071A (10 μM) observed by confocal microscopy in PEG8000 solution. The occupied areas of the droplets were quantified and shown. The GST-GFP-tagged recombinant proteins purified from bacterial cells were examined by CBB staining. (**G**) Representative micrographs and quantitative analysis of time-lapse opto-droplet formation of mCherry-Cry2-tagged proteins as indicated after blue light activation (n > 15). (**H**) Representative micrographs and quantitative analysis of the puncta intensity of PHF8 and mCherry-LacI-TOPBP1 in the presence of vehicle, auranofin (2 μM, 2 hr), and 1,6-hexanediol (1,6-HD, 2.5% for 5 min) as indicated. The occupied area of PHF8 and mCherry-marked puncta in U2OS cells stably integrated with 256 × LacO repeats was quantified and normalized to that of the corresponding nucleus (n > 20). (**I**) Representative micrographs and quantitative analysis of the puncta intensity of FANCJ and mCherry-LacI-TOPBP1 in the presence of vehicle, auranofin (2 μM, 2 hr), and 1,6-hexanediol (1,6-HD, 2.5% for 5 min) as indicated. The occupied area of FANCJ and mCherry-marked puncta in U2OS cells stably integrated with 256 × LacO repeats was quantified and normalized to that of the corresponding nucleus (n > 17). (**J**) Immunoblotting analysis of the expression of the indicated proteins with cellular extracts from (**I**). (**K**) Representative micrographs and quantitative analysis of the puncta intensity of mCherry-LacI-TOPBP1 and mCherry-LacI in U2OS cells expressing the indicated siRNAs. The occupied area of mCherry-marked puncta was quantified and normalized to that of the corresponding nucleus (n ⩾ 15). (**L**) Immunoblotting analysis of the knockdown effects using cellular extracts from (**K**).
Data are mean ± SDs for (**F-I**) and (**K**) from biological triplicate experiments. $^{**}P < 0.01$; NS, not significant; one-way ANOVA followed by Tukey's multiple comparisons test for (**F**), (**H-I**), and (**K**); two-way ANOVA for (**G**). Scale bars, 10 μm.

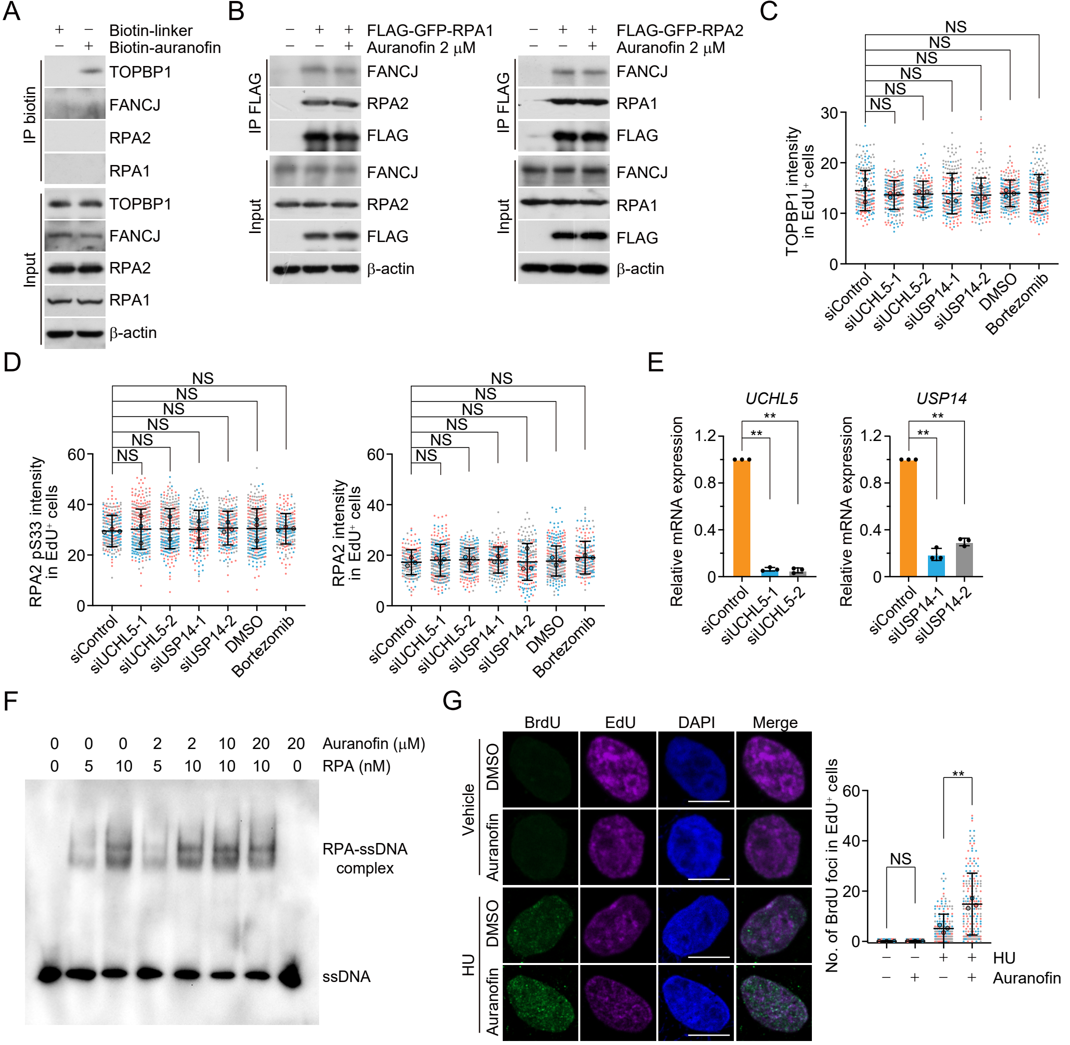

Supplemental Figure 5 (Figure 5 continued). Auranofin prevents RPA loading to perturbed replication forks. (**A**) Pull-down analysis of the interaction between biotin-auranofin and the indicated proteins. (**B**) Co-IP analysis of the interaction of FLAG-GFP-tagged RPA1 or RPA2 with FANCJ using cellular extracts from HeLa cells treated by auranofin (2 μM, 2 hr). (**C-D**) Immunostaining and confocal microscopy analysis of TOPBP1 (**C**), RPA2 (**D**), and RPA2 pS33 (**D**) foci formation. Cells were transfected with siRNAs as indicated or treated with bortezomib (10 μM) for 4 hr before collection. Replication stress was induced by CPT (2 μM) for 1 hr, and the actively replicating cells were labeled by EdU (10 μM) for 1 hr. Cells were then pre-extracted and fixed. The intensity of foci in EdU-positive cells was quantified and shown (n > 55). (**E**) Examination of the siRNA-mediated knockdown effect of UCHL5 and USP14 by reverse transcription coupled quantitative PCR. (**F**) Analysis of ssDNA-RPA binding in the absence or presence of auranofin. Gel shift assays were performed with 5' biotin-labeled 34-nt ssDNA (10 nM) and an increasing amount of RPA in the absence or presence of different doses of auranofin followed by electrophoresis and visualization. Free and bound ssDNA is marked as indicated. (**G**) Immunostaining and confocal microscopy analysis of BrdU foci formation in auranofin-treated U2OS cells (2 μM, 2 hr). Replication stress was induced by HU (1 mM) for 4 hr, and the actively replicating cells were labeled by EdU (10 μM) for 1 hr before collection. Cells were then pre-extracted and fixed. The intensity of foci in EdU-positive cells was quantified and shown (n > 46).

Data are mean ± SDs for (**C-E**) and (**G**) from biological triplicate experiments. **$P < 0.01$; NS, not significant; one-way ANOVA followed by Tukey's multiple comparisons test for (**C-E**) and (**G**).

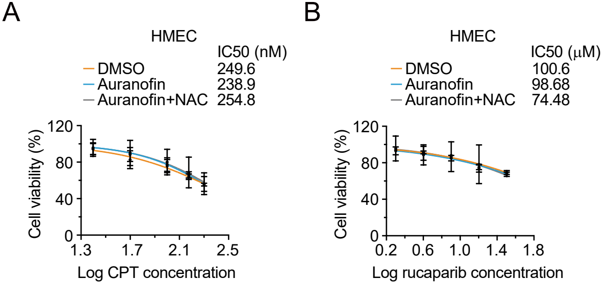

Supplemental Figure 6 (Figure 6 continued). Survival analysis of the synthetic lethality of auranofin and CPT (A) or rucaparib (B) in HMECs. Auranofin at a concentration of 1.5 μM was used. Data are mean ± SDs from one representative data of biological triplicate experiments. NS, not significant; two-way ANOVA.

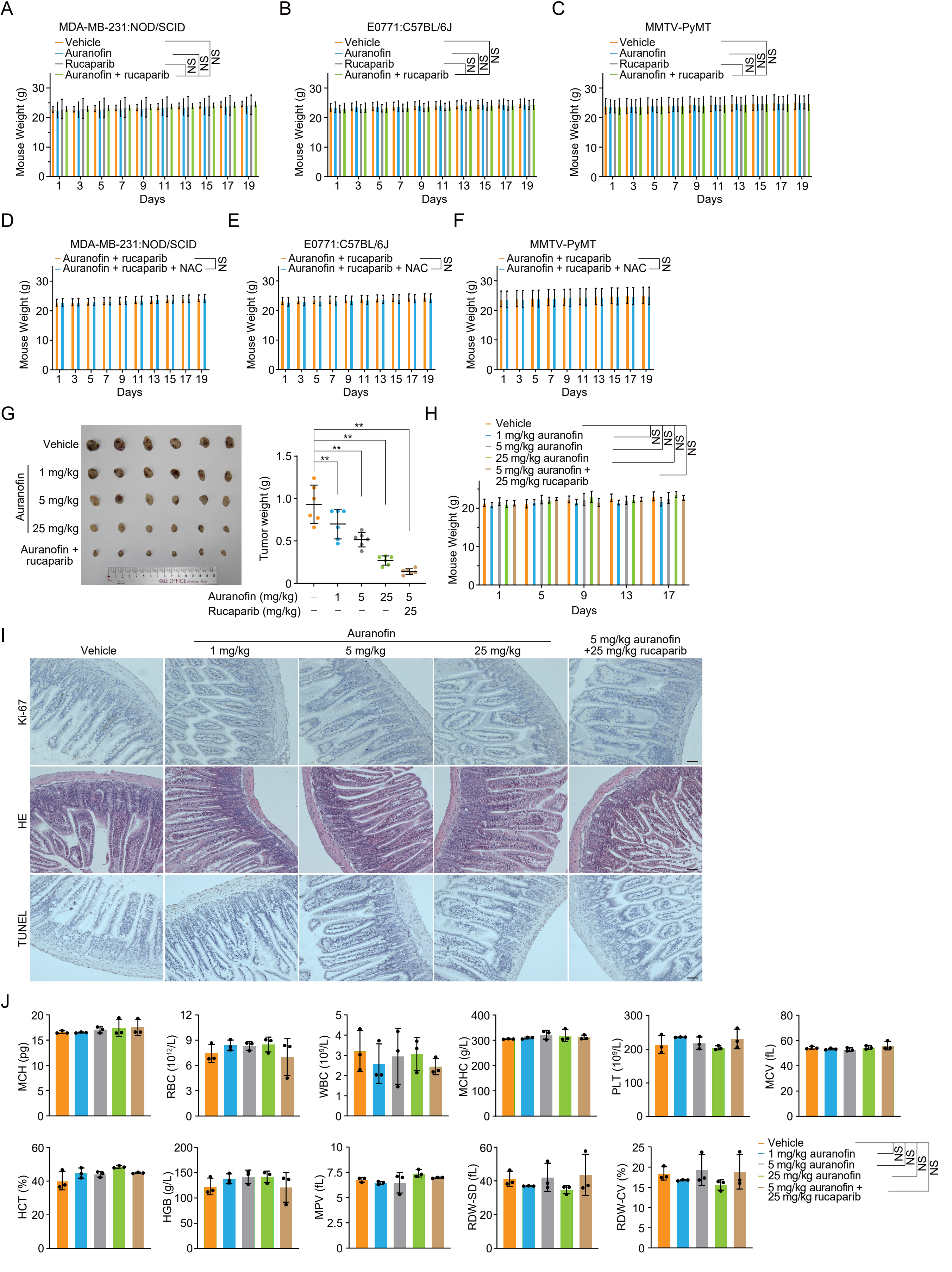

A
MDA-MB-231:NOD/SCID
Vehicle
Auranofin
Rucaparib
Auranofin + rucaparib
NS
Mouse Weight (g)
Days
B
E0771:C57BL/6J
C
MMTV-PyMT
D
E
F
Auranofin + rucaparib + NAC
G
Vehicle
Auranofin
1 mg/kg
5 mg/kg
25 mg/kg
Auranofin + rucaparib
Tumor weight (g)
Auranofin (mg/kg)
Rucaparib (mg/kg)
**
H
1 mg/kg auranofin
5 mg/kg auranofin
25 mg/kg auranofin
5 mg/kg auranofin + 25 mg/kg rucaparib
I
5 mg/kg auranofin +25 mg/kg rucaparib
Ki-67
HE
TUNEL
J
MCH (pg)
RBC (10^{12}/L)
WBC (10^9/L)
MCHC (g/L)
PLT (10^9/L)
MCV (fL)
HCT (%)
HGB (g/L)
MPV (fL)
RDW-SD (fL)
RDW-CV (%)

Supplemental Figure 7 (Figure 7 continued). Breast tumor is synthetically susceptible to the action of auranofin and rucaparib. (**A**) Mouse weight of mice bearing xenografts from MDA-MB-231 cells. NOD/SCID mice carrying tumors were treated with rucaparib, auranofin, or both every two days. (**B**) Mouse weight of mice bearing xenografts from E0771 cells. C57BL/6J mice carrying tumors were treated with rucaparib, auranofin, or both every two days. (**C**) Mouse weight of mice bearing genetically engineered mammary gland tumors. PyMT mice carrying tumors were treated with rucaparib, auranofin, or both every two days. (**D-F**) Mouse weight of mice bearing tumors of MDA-MB-231 cells (**D**), E0771 cells (**E**), and genetically engineered mammary gland tumors (**F**) under combinatorial auranofin and rucaparib treatment in the absence or presence of NAC. (**G**) Tumor size and weight of xenografts from E0771 cells. C57BL/6J mice carrying tumors were treated with vehicle, different doses of auranofin (1, 5, 25 mg/kg), or both auranofin (5 mg/kg) and rucaparib (25 mg/kg) every two days. (**H**) Mouse weight of mice bearing tumor xenografts from (**G**). (**I**) Ki-67, HE, and TUNEL staining of intestinal tissues using mice from (**G**). Representative images from 3 mice in each treatment are shown. Scale bars, 100 μm. (**J**) Quantification of key complete blood count (CBC) parameters of C57BL/6J mice used in (**G**). RBC, red blood cell count; WBC, white blood cell count; MCHC, mean corpuscular hemoglobin concentration; PLT, platelet count; MCV, mean corpuscular volume; MCH, mean corpuscular hemoglobin; HCT, hematocrit; HGB, hemoglobin; MPV, mean platelet volume; RDW-SD, red cell distribution width-standard deviation; RDW-CV, red cell distribution width-coefficient of variation.

Data are mean ± SDs for (**A-H**) and (**J**). $^{**}P < 0.01$; NS, not significant; two-way ANOVA for (**A-F**) and (**H**), one-way ANOVA followed by Tukey's multiple comparisons test for (**G**) and (**J**).

Supplemental Table 2. epegRNA and sgRNA sequence.

Guide RNAs	Sequences
epegRNA	caccGTGGATCAGTCTCGAGAAGCgttttAGAGCTAGAAATAGCAAGTTAA AATAAGGCTAGTCCGTTATCAACTTGAAAAAGTGGCACCGAGTCGgt gcGTTTGGCGCCTGCTTCTCGAGACTAAATTTGT
nicking sgRNA	caccgAGAACTCATCATAGCAATCT

Supplemental Table 3. siRNA sequences.

siRNAs	Sequences
siTOPBP1	GCACAAGGUUUAAUGAGGA
siRPA1	GAGAAUCAGUGGGUGACUU
siUCHL5-1	GGGUCUUCACCGAGCUCAUUA
siUCHL5-2	AGCCAGUUCAUGGGUUAAUUU
siUSP14-1	CGCAGAGUUGAAAUAAUGGAA
siUSP14-2	CCCAAGAUUCAGCAGUCAGAU
siPHF8-1	CGAAUUUGACUUGGAUUCA
siPHF8-2	UGGGAGUGUUAGUAAUCAA
siFANCJ-1	AGCUUACCCGUCACAGCUU
siFANCJ-2	GCUAAGAAACAGGCAUCCAUA

Supplemental Table 4. shRNA sequences.

shRNAs	Sequences
shTOPBP1-1	TGTAAATATCTGAAGCTGTATTCAAGAGATACAGCTTCAGATA TTTACTTTTTTC
shTOPBP1-2	TGCACAAGGTTTAATGAGGATTCAAGAGATCCTCATTAAACCT TGTGCTTTTTTC
shTrxR1-1	TCGTCAAGAGATAACAACAAATTTCAAGAGAATTTGTTGTTAT CTCTTGACGTTTTTTC
shTrxR1-2	TCCTGCAAGACTCTCGAAATTATTCAAGAGATAATTTCGAGAG TCTTGCAGGTTTTTTC

Supplemental Table 5. qRT-PCR and genome editing PCR primers.

Targets	Sequences
TOPBP1 (F1411A)	5': GGATTCACGTTTCAGTGTTCTGG;<br>3': GTTCTCAAGCAGTACACGTTC
UCHL5	5': GAGTGGTGCCTCATGGAAAG;<br>3': CAAGTCGGGAGTCCTGAACC
USP14	5': ATGCCGCTCTACTCCGTTACT;<br>3': GCCTTGAATACCATTGGAGGTTC
