# Supplementary material for: Auranofin attenuates TOPBP1-mediated ATR replication stress response and improves chemotherapeutic response in breast tumor models
Source: J Clin Invest. 2025 Dec 15;135(24):e180106. doi: 10.1172/JCI180106 (PMC12700547; doi:10.1172/JCI180106)

Full unedited gel for Figure 2B

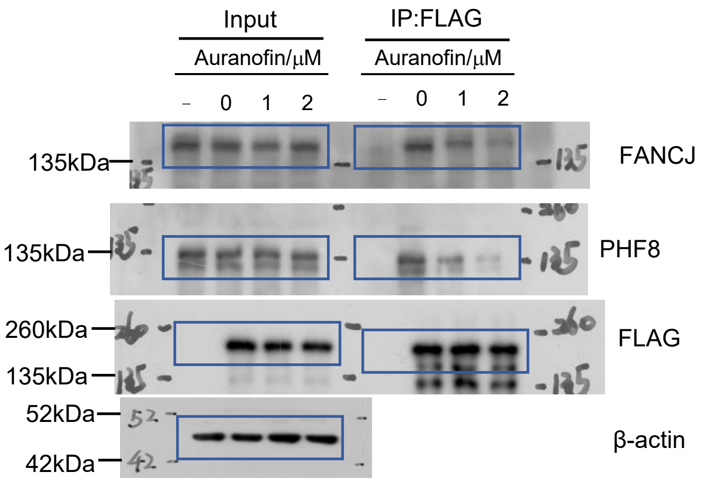

Full unedited gel for Figure 2C

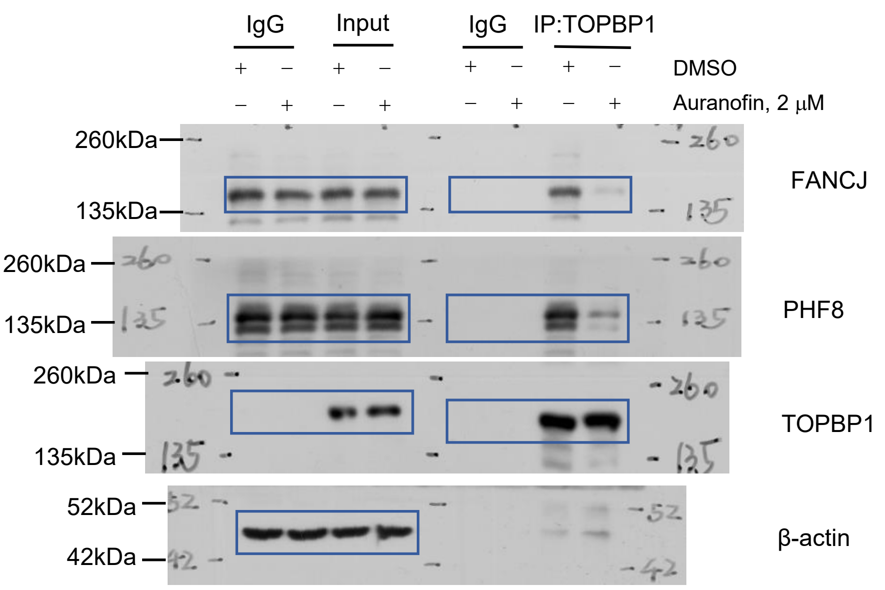

Full unedited gel for Figure 2D

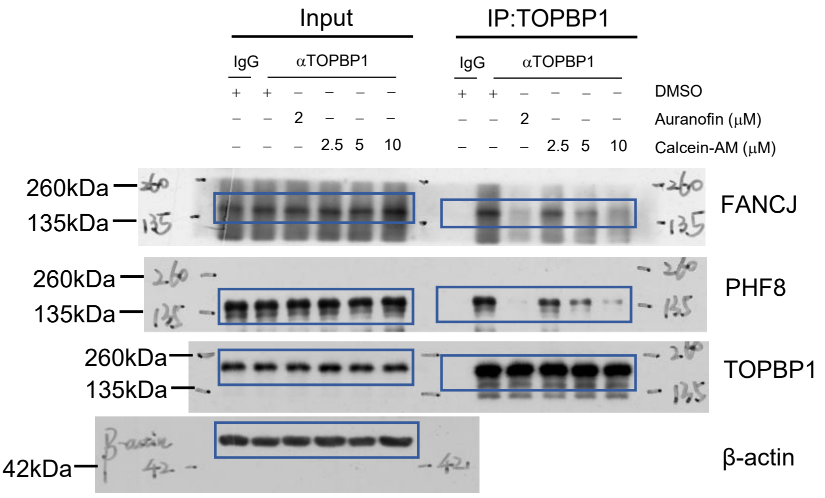

Full unedited gel for Figure 2E

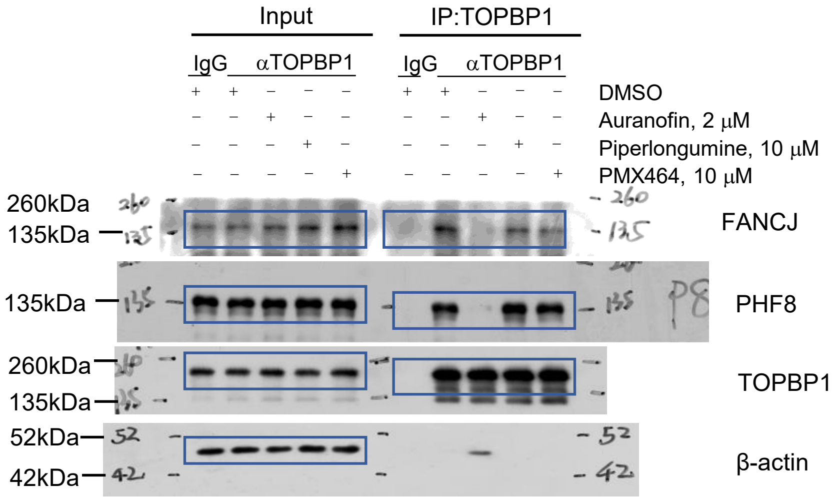

Full unedited gel for Figure 2I

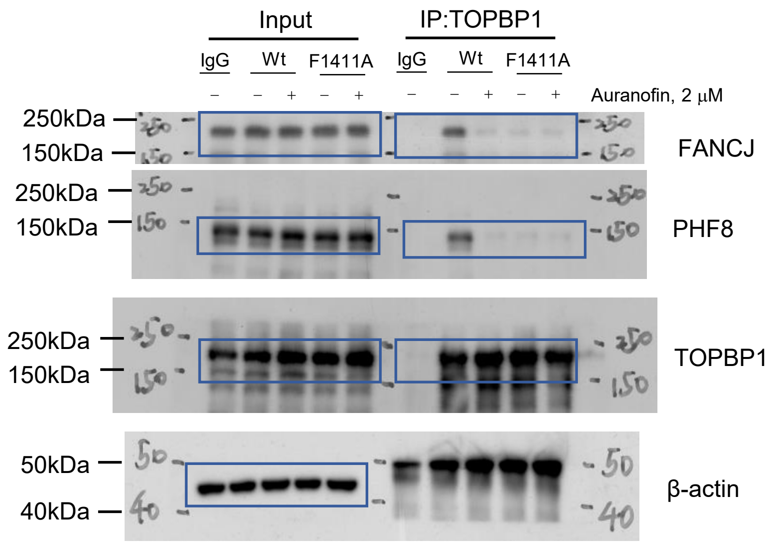

Full unedited gel for Figure 3B

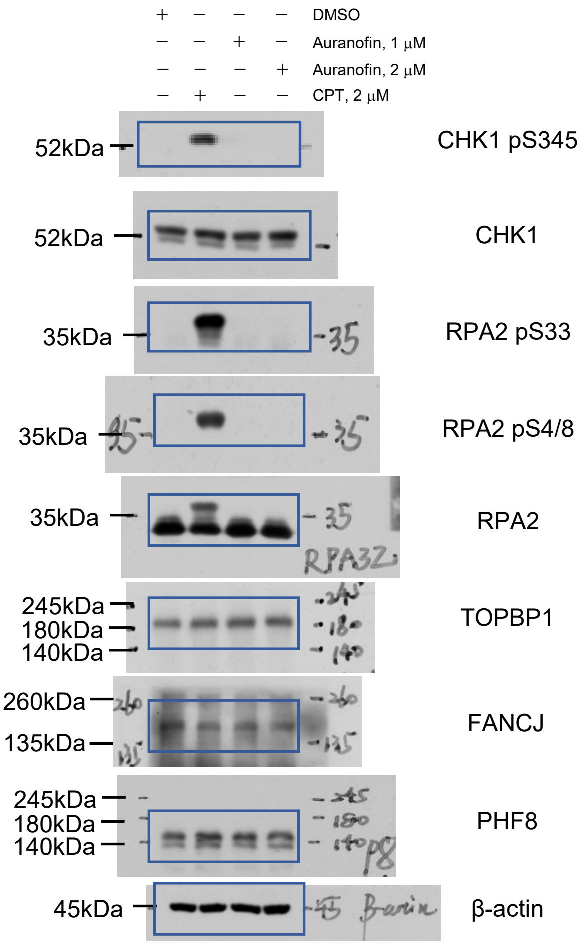

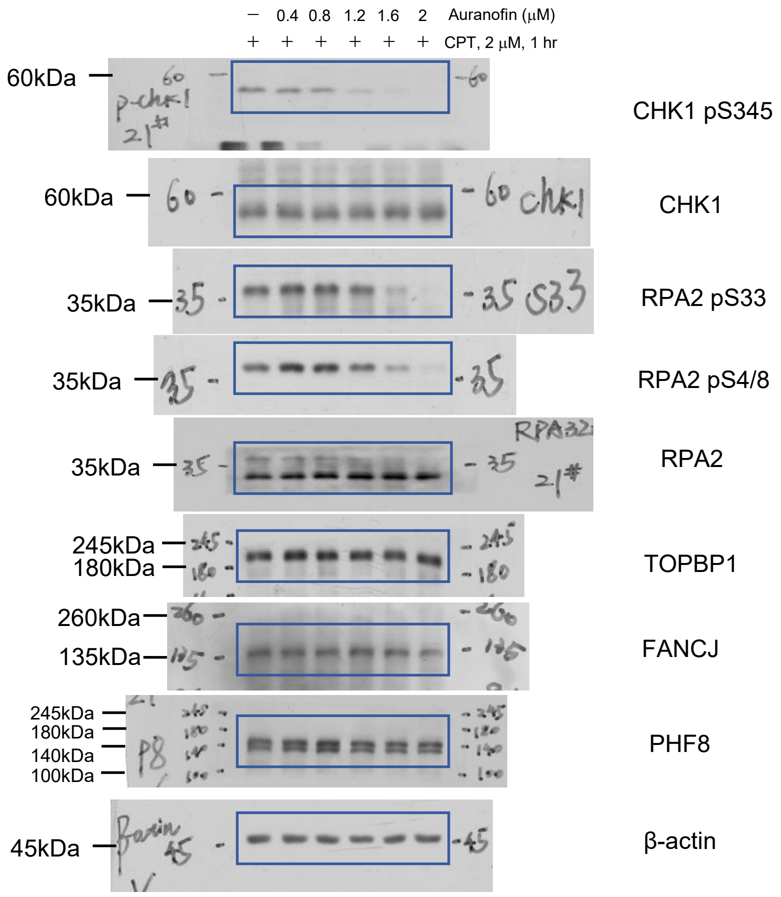

Full unedited gel for Figure 3C

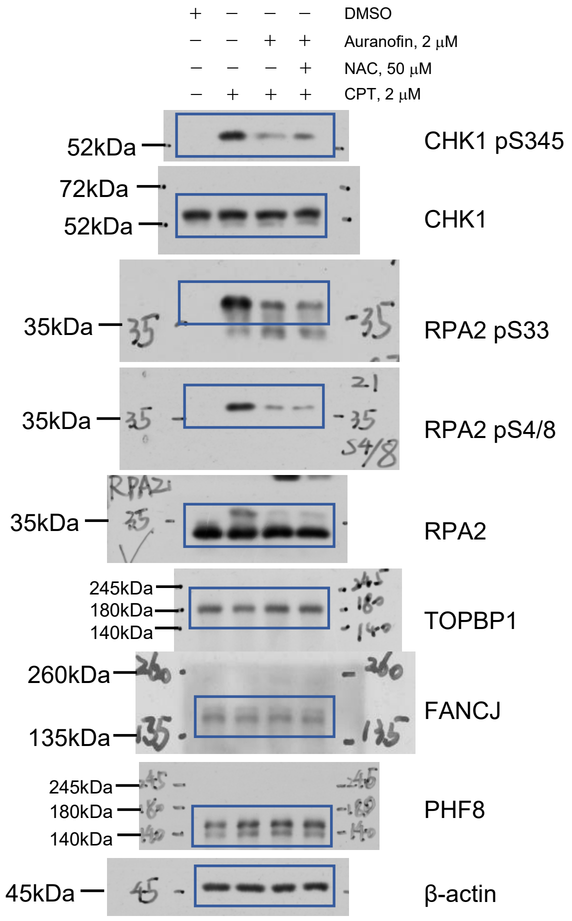

Full unedited gel for Figure 3D

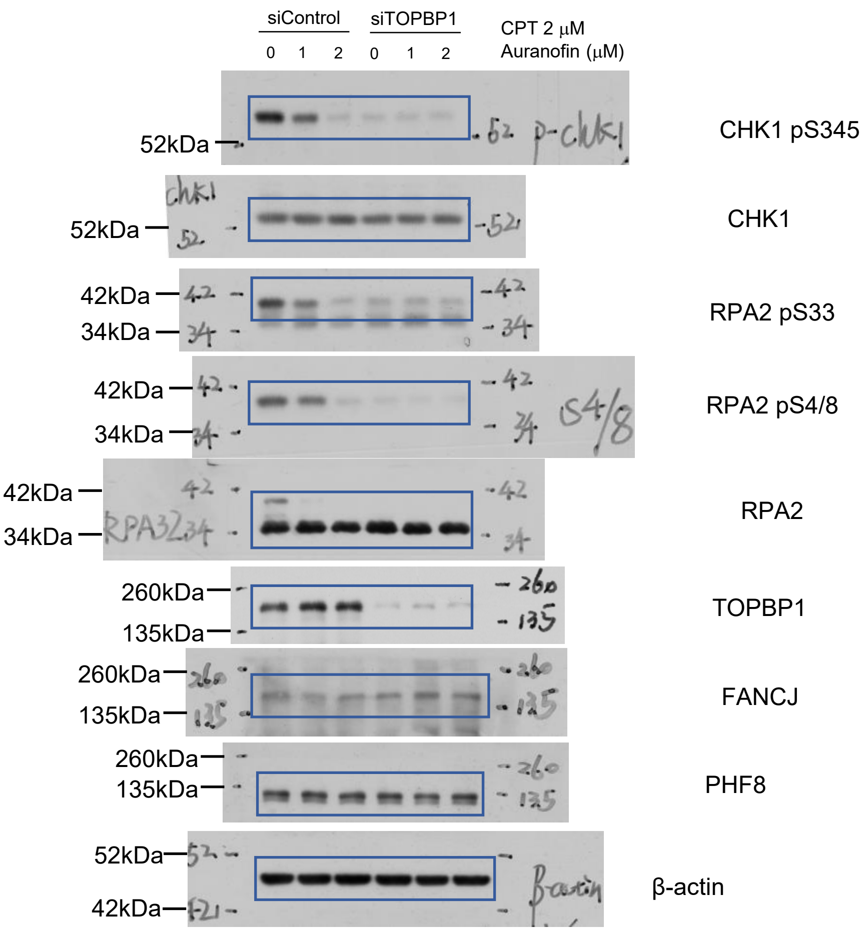

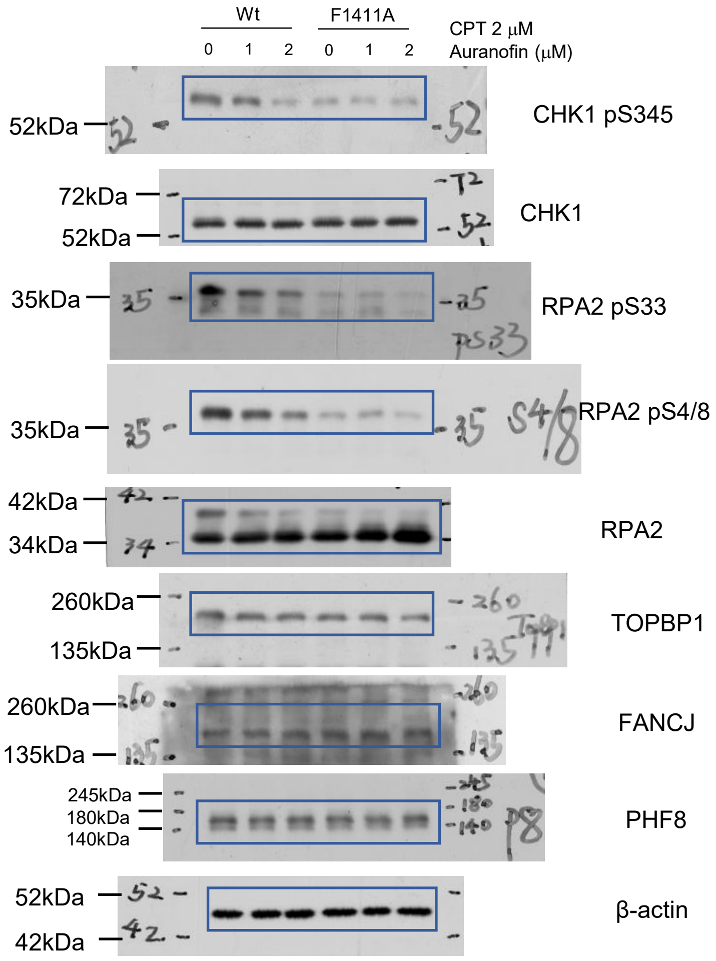

Full unedited gel for Figure 4A

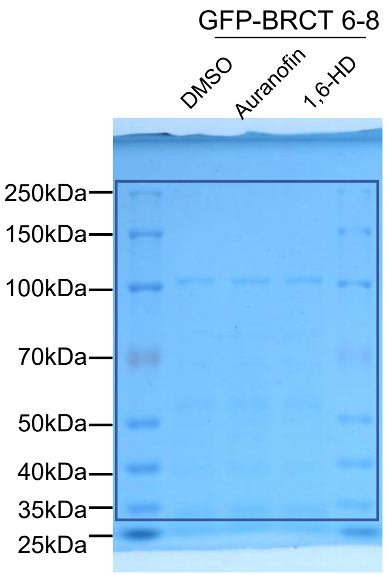

Full unedited gel for Figure 4B

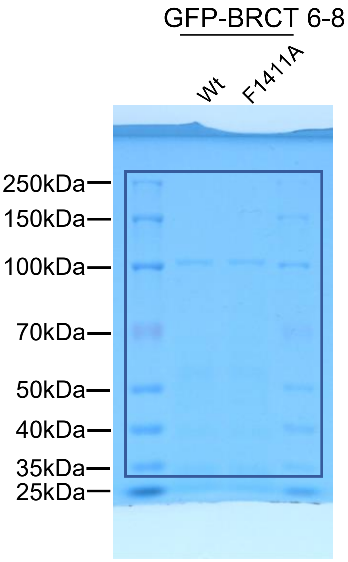

Full unedited gel for Figure 5C

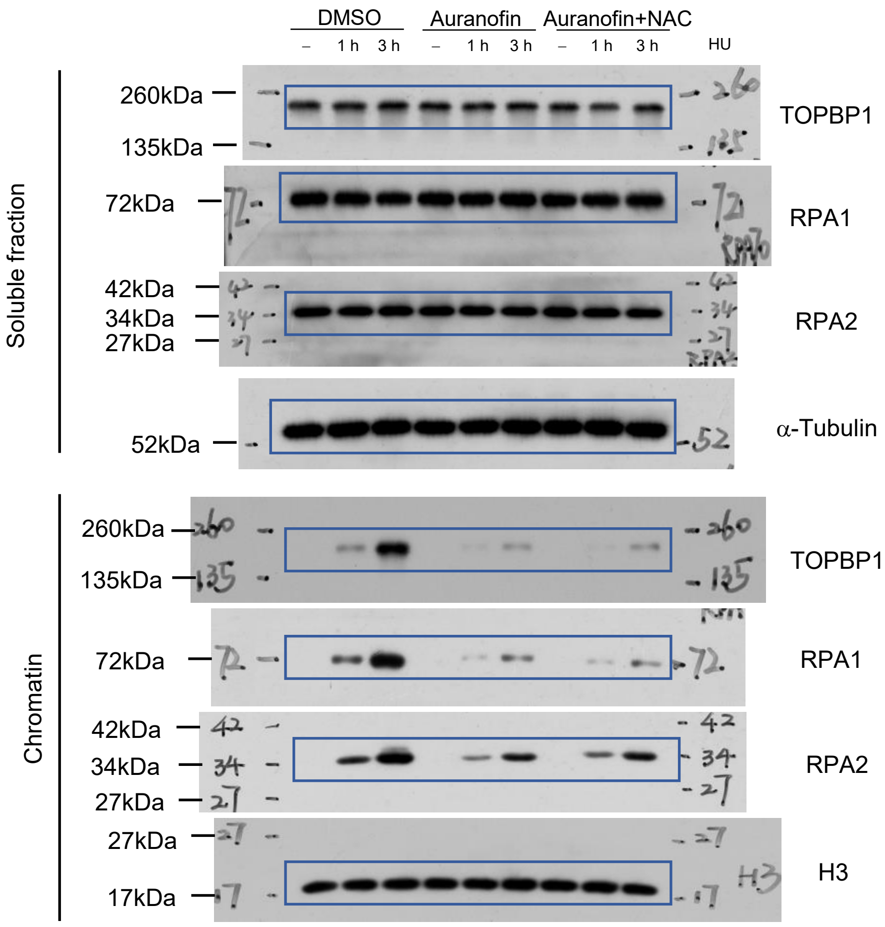

Full unedited gel for Figure 5E

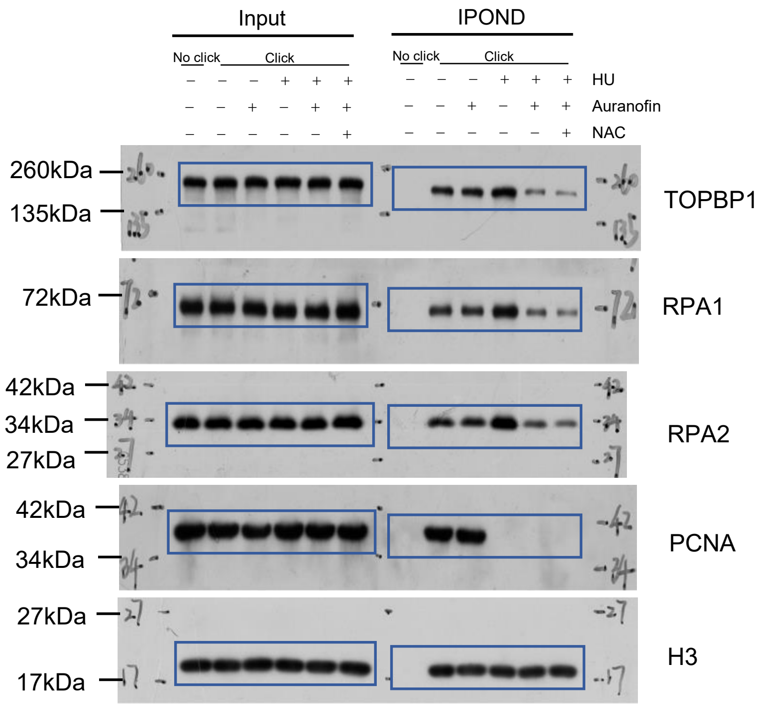

Full unedited gel for Figure 6A

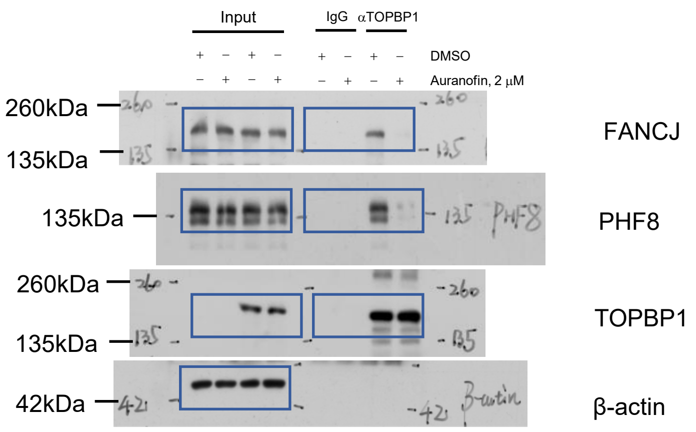

Full unedited gel for Figure 6B

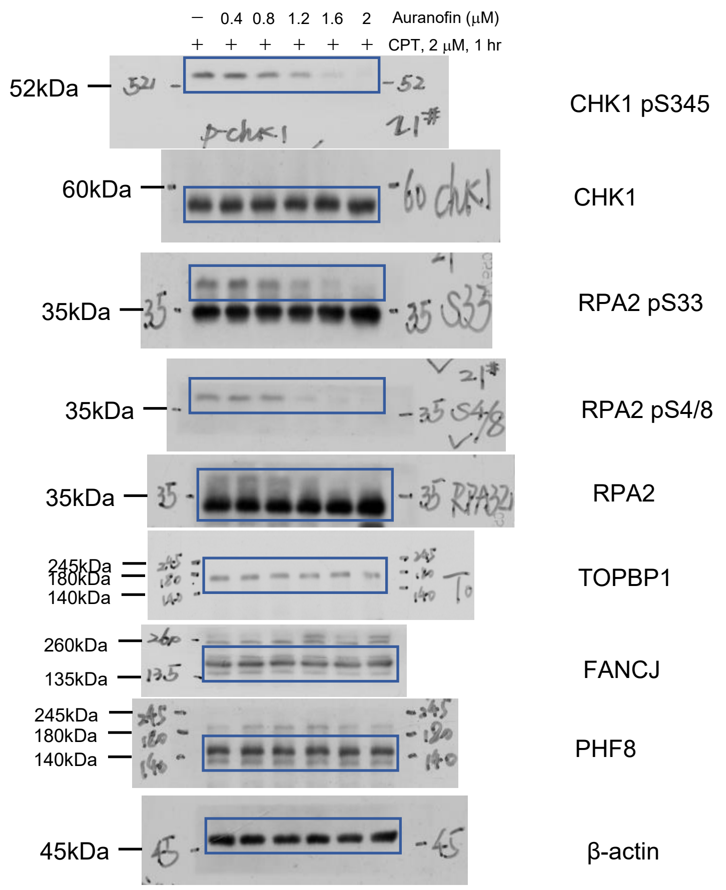

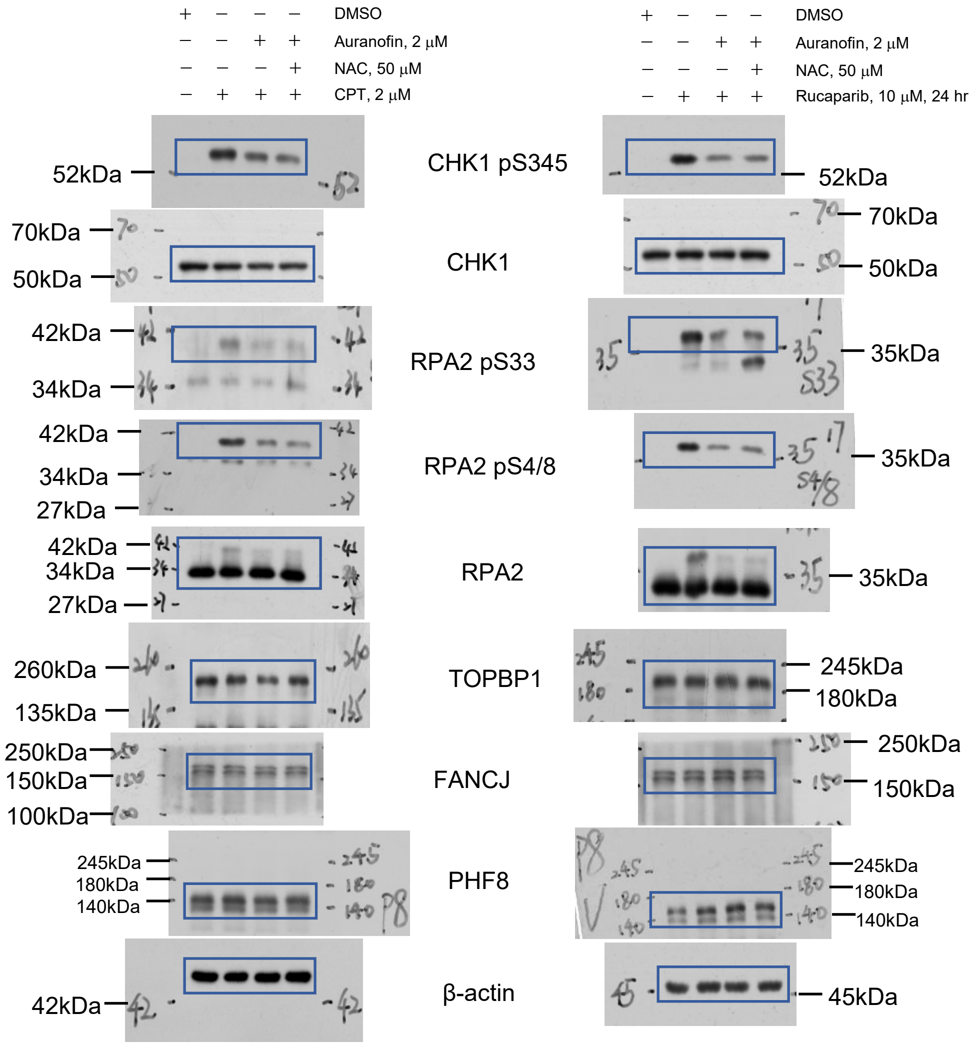

Full unedited gel for Figure 6G

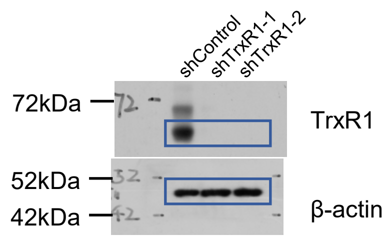

Full unedited gel for Figure 6H

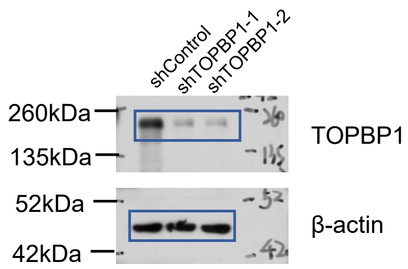

Full unedited gel for Figure S2A

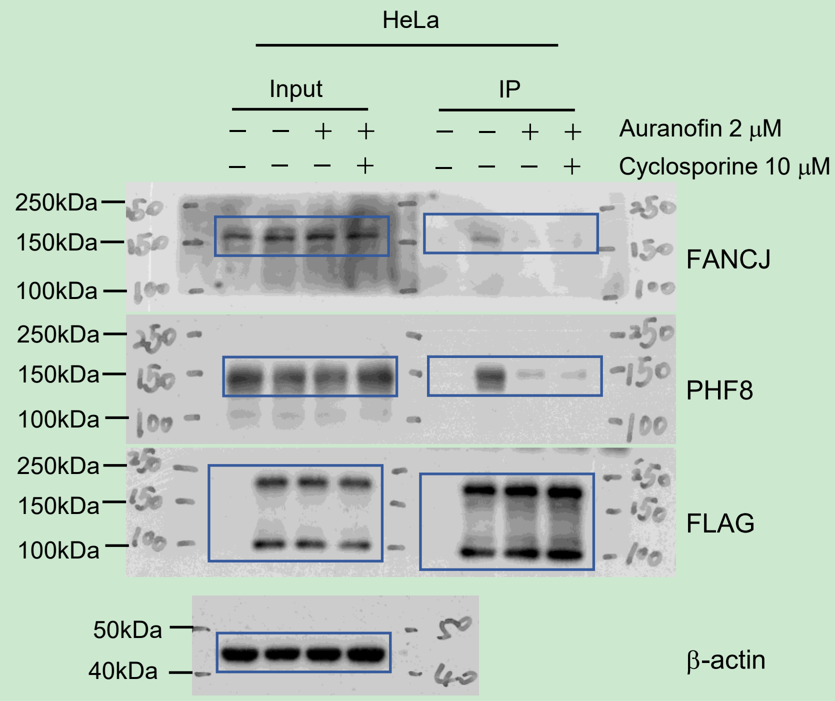

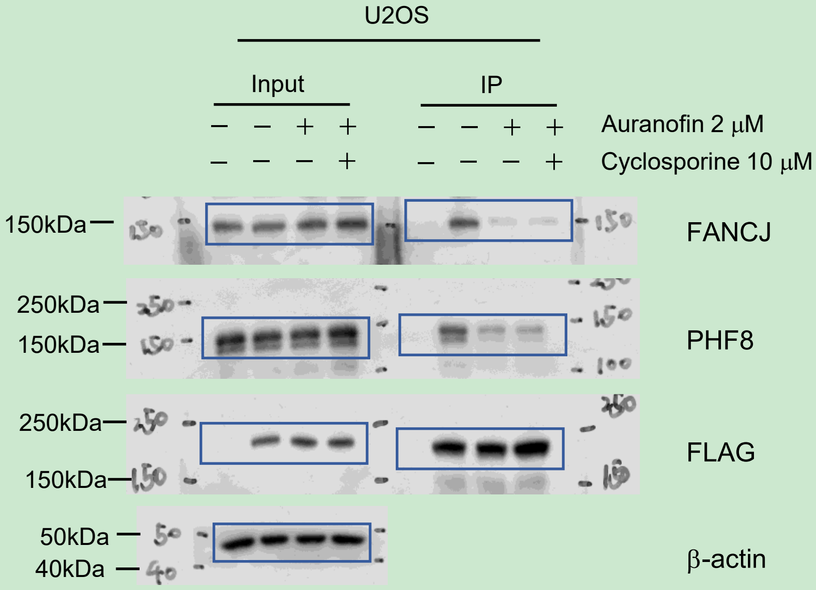

Full unedited gel for Figure S2B

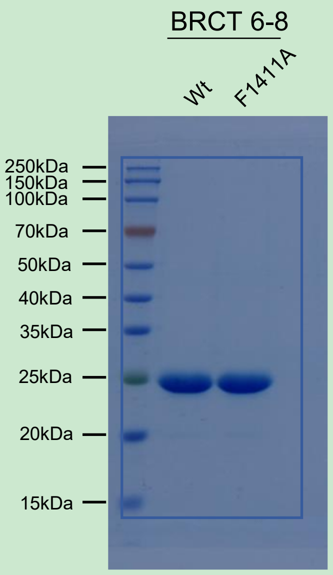

Full unedited gel for Figure S2C

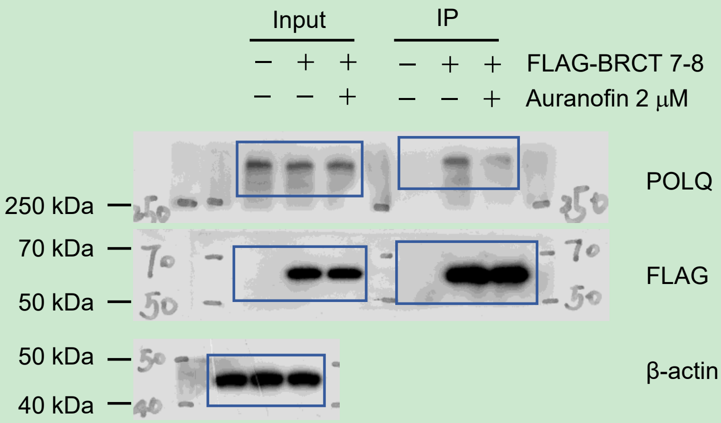

Full unedited gel for Figure S2F

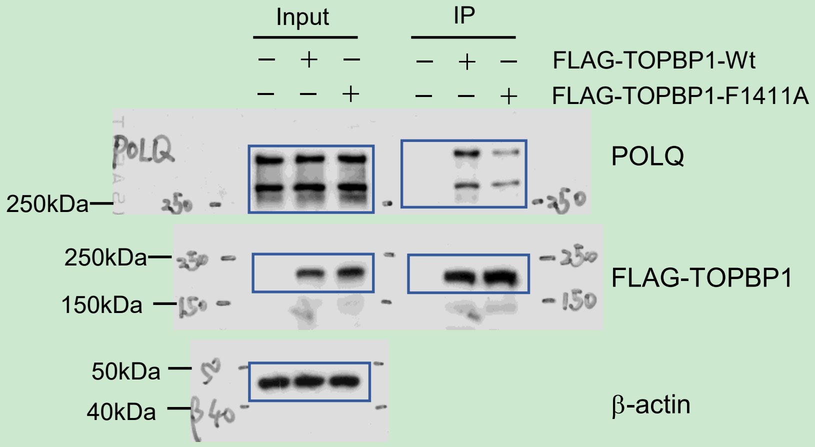

Full unedited gel for Figure S2G

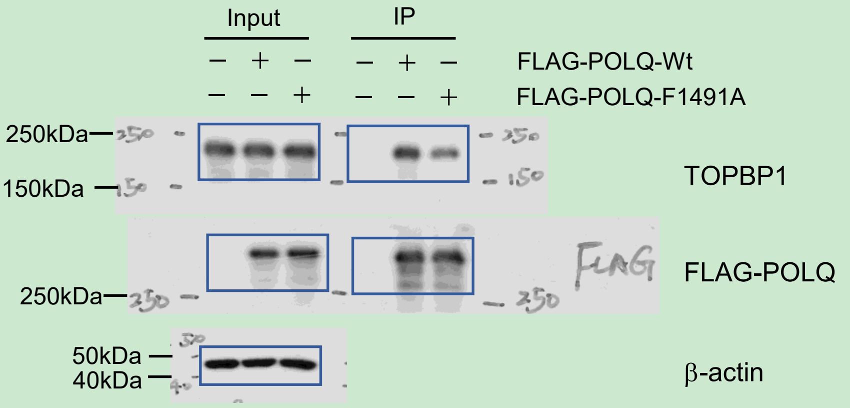

Full unedited gel for Figure S2I

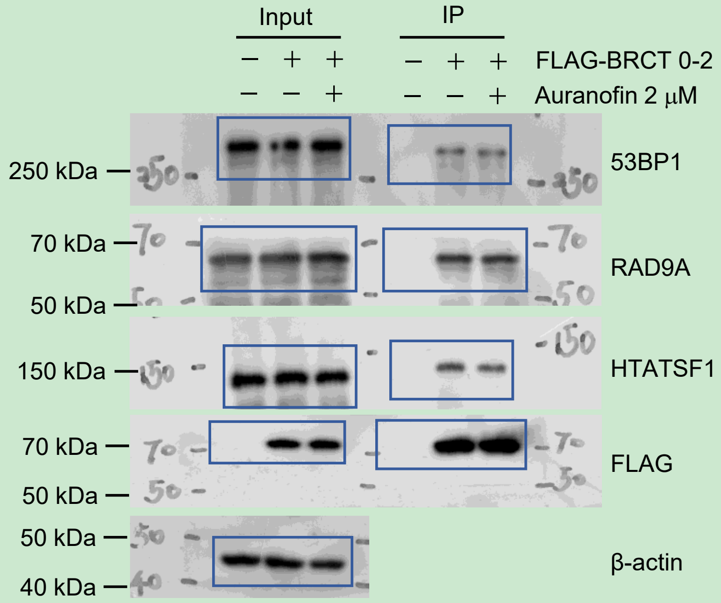

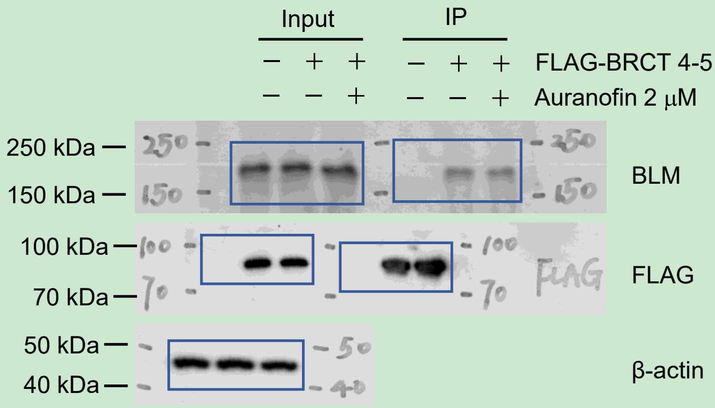

Full unedited gel for Figure S2J

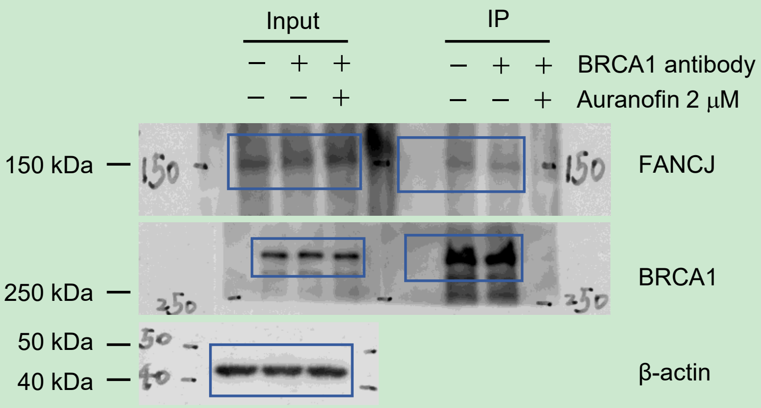

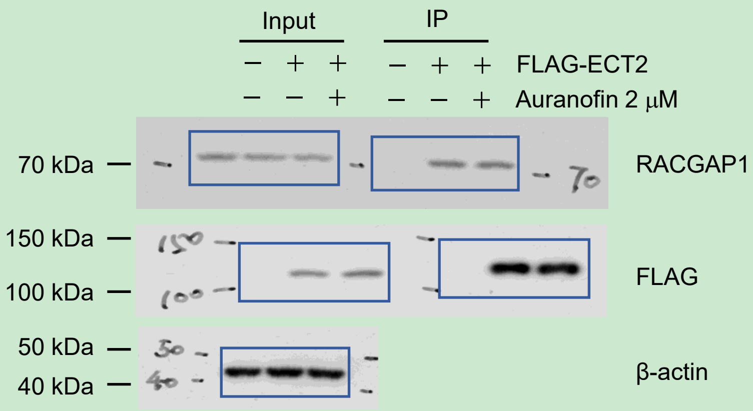

Full unedited gel for Figure S2L

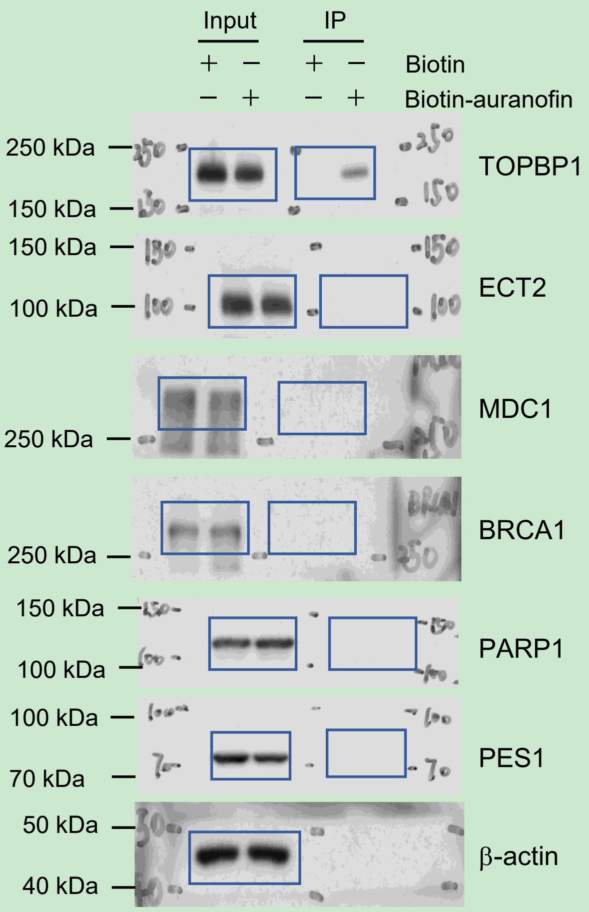

Full unedited gel for Figure S4F

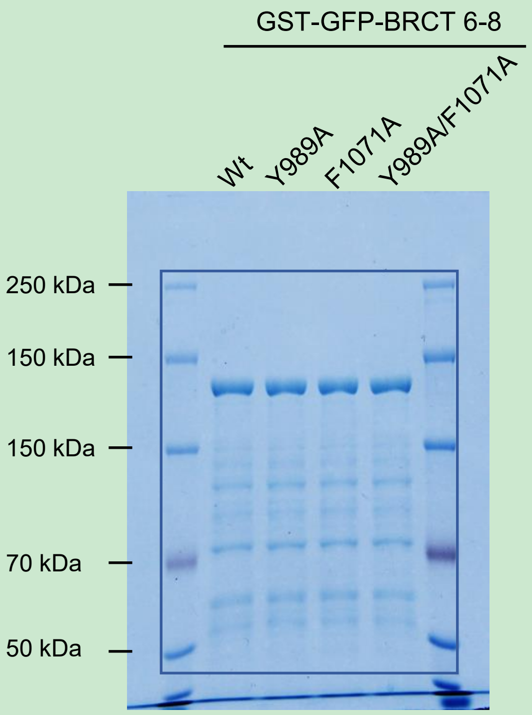

Full unedited gel for Figure S4J

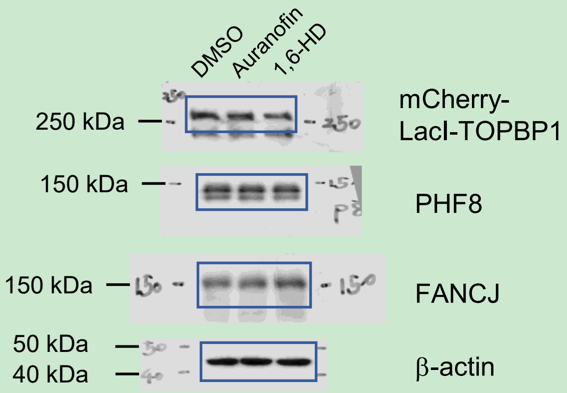

Full unedited gel for Figure S4L

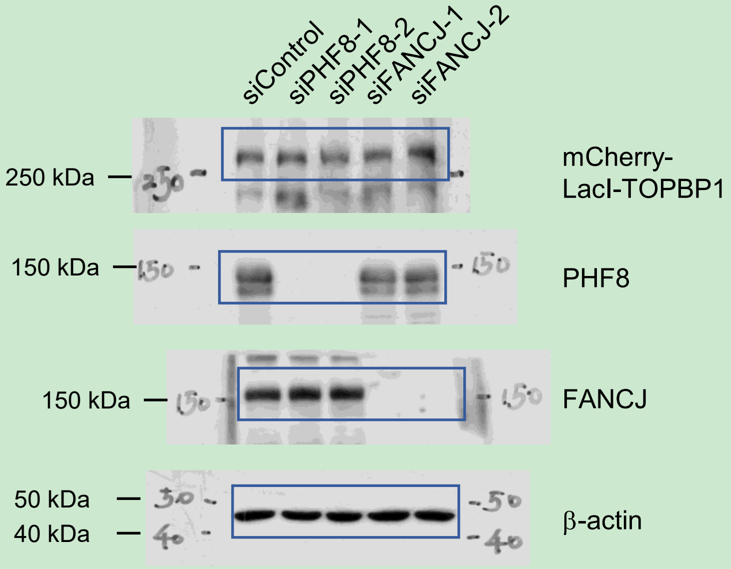

Full unedited gel for Figure S5A

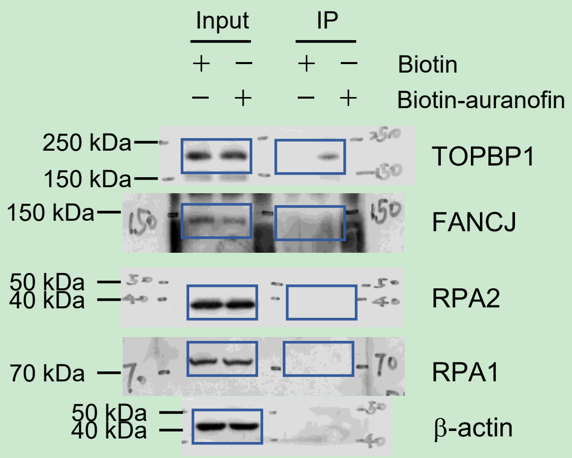

Full unedited gel for Figure S5B

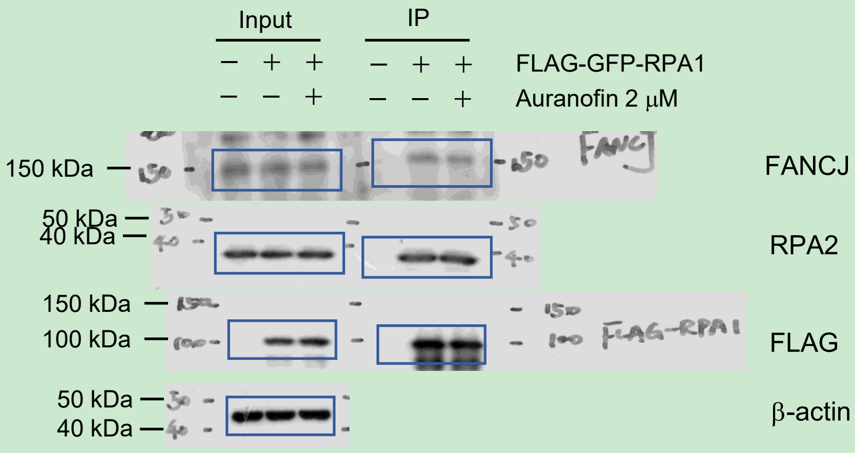

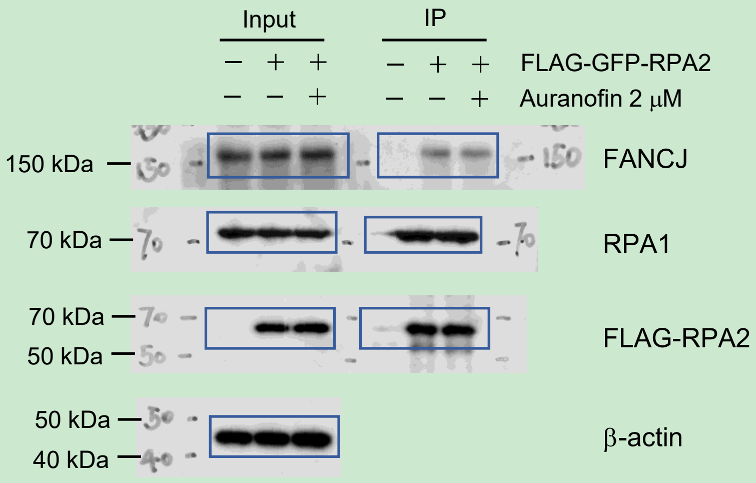

Supplement: Unedited blot and gel images [file jci-135-180106-s114.pdf]
